# Supplementary material for: Fostering Success and Promoting Professional Development of Clinician Educator Mentees: A Workshop for Mentors
Source: MedEdPORTAL. 2023 Jun 27;19:11321. doi: 10.15766/mep_2374-8265.11321 (PMC10293477; doi:10.15766/mep_2374-8265.11321)
Supplement: Supplementary file 1 — CE Training Workshop.pptxFacilitator Guide.docxIndividual Development and Mentoring Plans.docxCase Studies.docxResource Guide.docxWorkshop Evaluation.docx [file mep_2374-8265.11321-s001.zip › A. CE Training Workshop.pptx]

## Slide 1
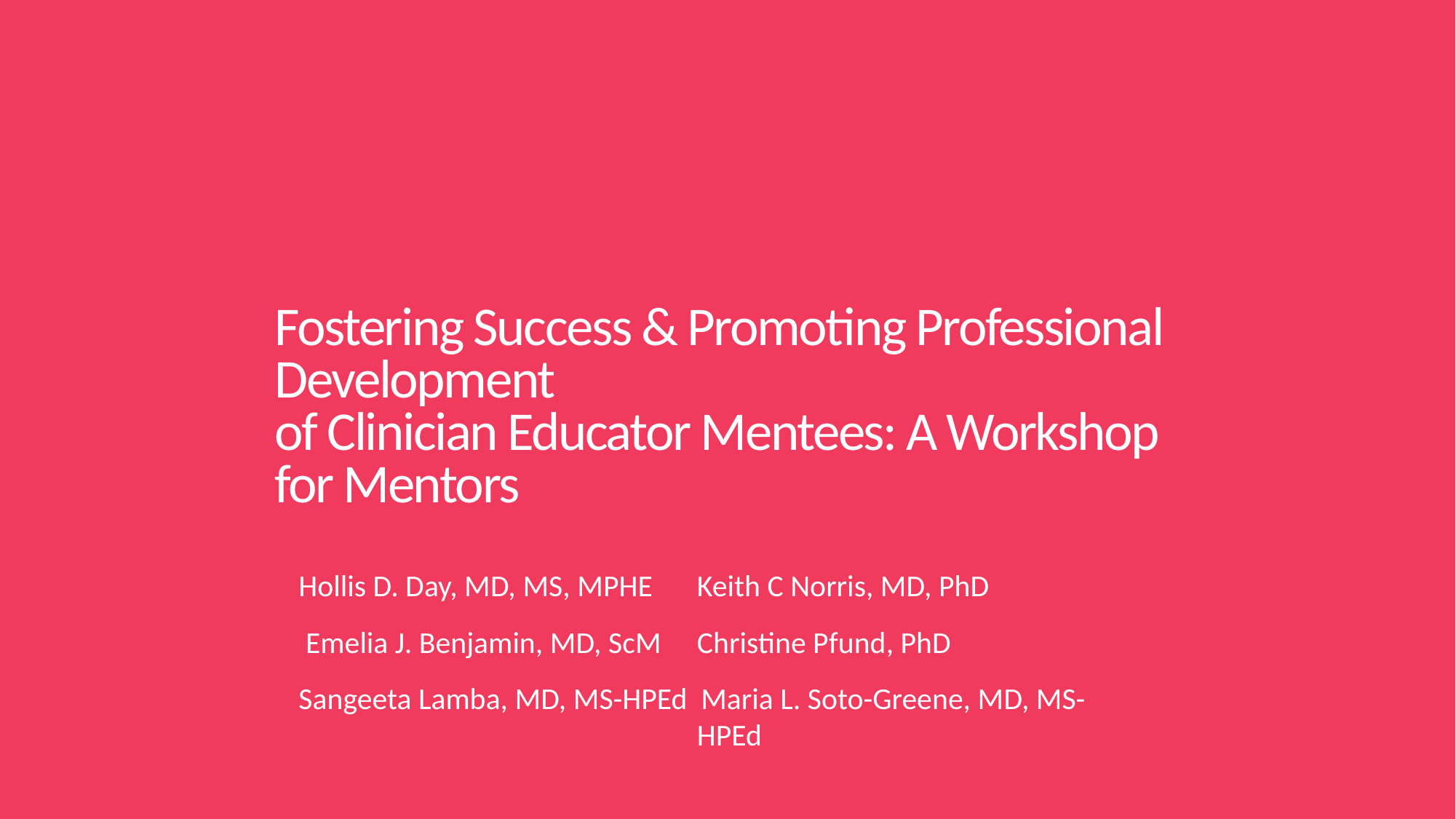

# Fostering Success & Promoting Professional Development
of Clinician Educator Mentees: A Workshop for Mentors
Hollis D. Day, MD, MS, MPHE
 Emelia J. Benjamin, MD, ScM
Sangeeta Lamba, MD, MS-HPEd
Keith C Norris, MD, PhD
Christine Pfund, PhD
 Maria L. Soto-Greene, MD, MS-HPEd

## Slide 2
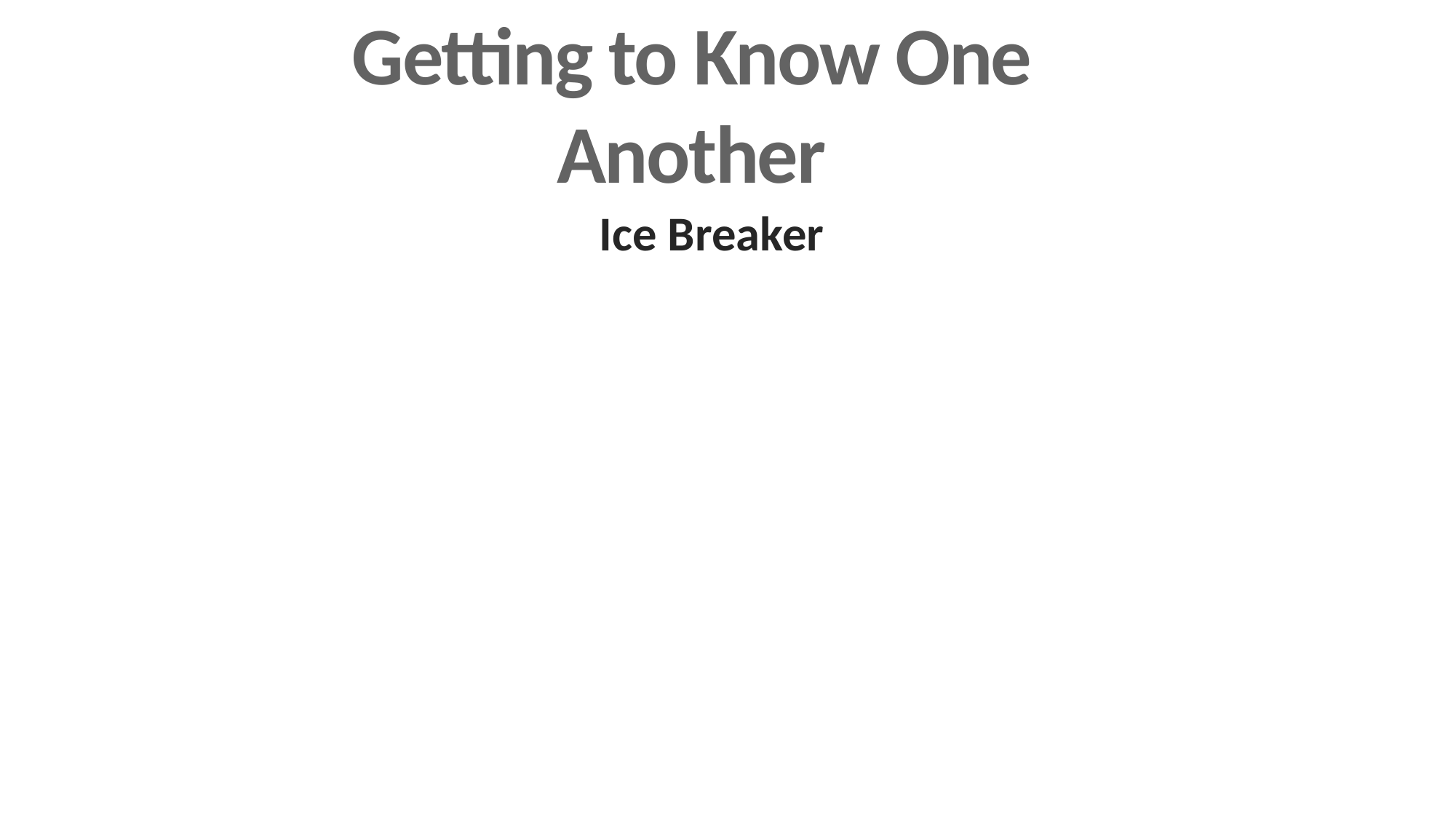

# Getting to Know One Another
Ice Breaker

## Slide 3
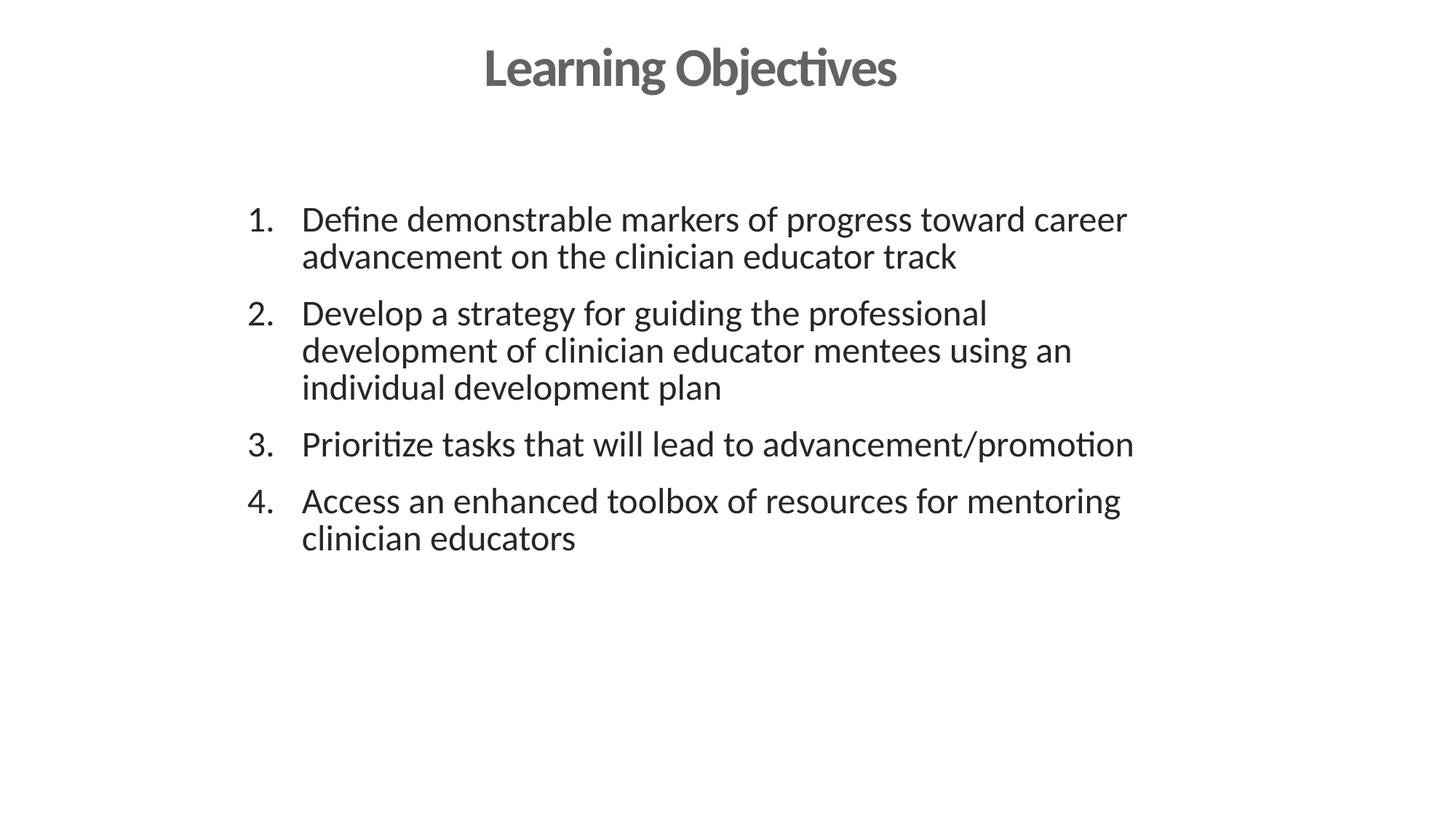

# Learning Objectives
Define demonstrable markers of progress toward career advancement on the clinician educator track
Develop a strategy for guiding the professional development of clinician educator mentees using an individual development plan
Prioritize tasks that will lead to advancement/promotion
Access an enhanced toolbox of resources for mentoring clinician educators

## Slide 4
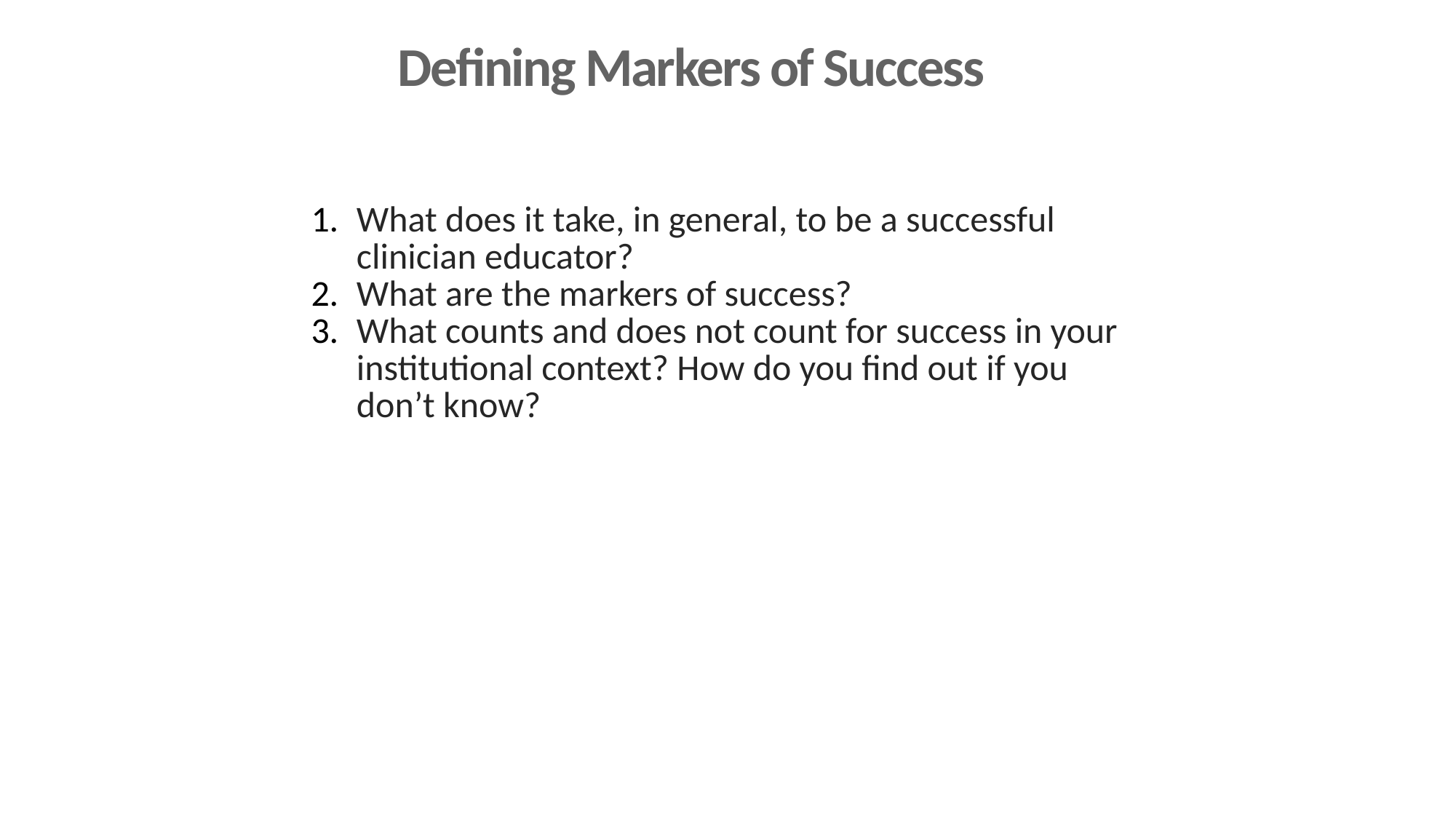

# Defining Markers of Success
What does it take, in general, to be a successful clinician educator?
What are the markers of success?
What counts and does not count for success in your institutional context? How do you find out if you don’t know?

## Slide 5
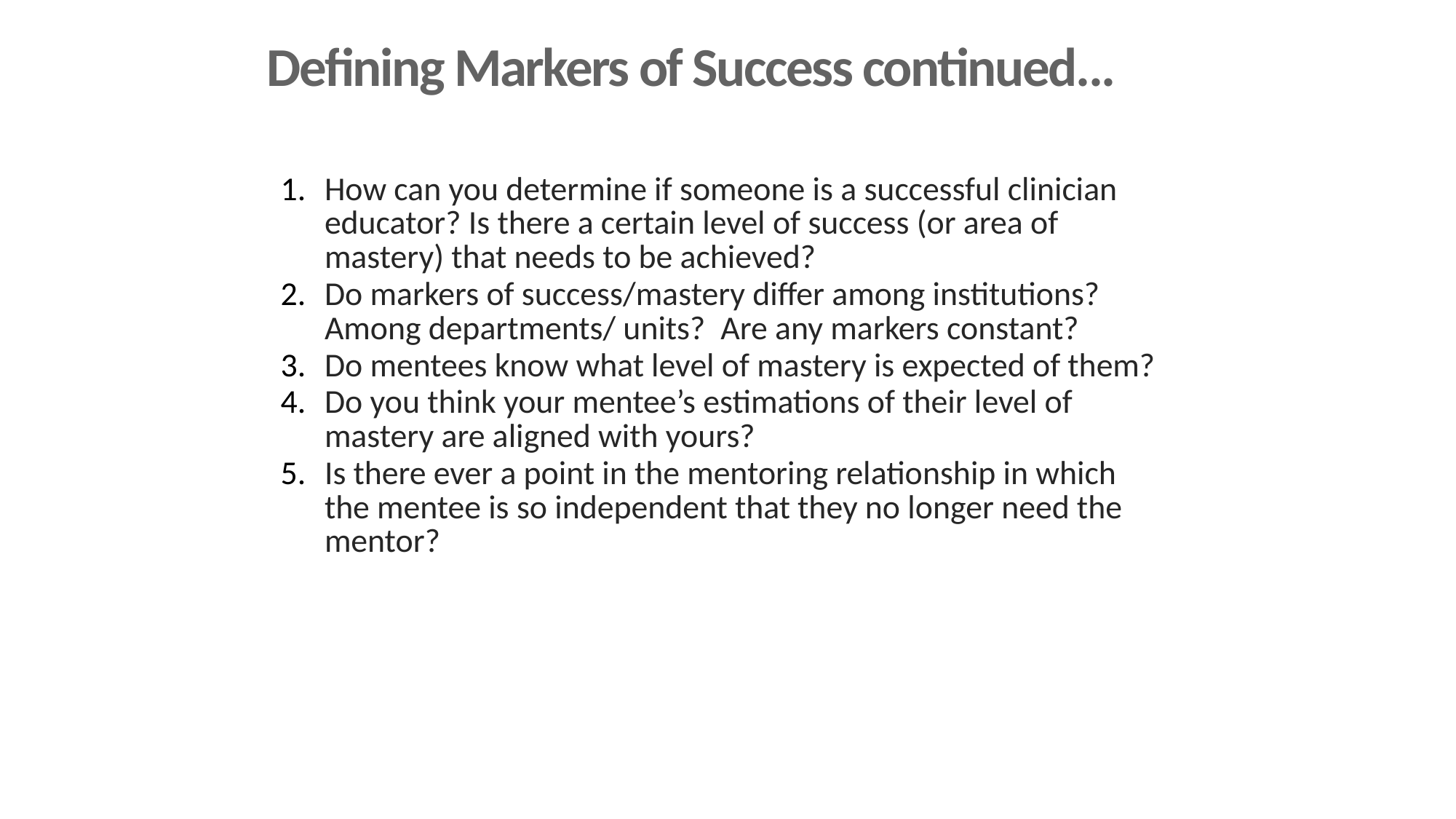

# Defining Markers of Success continued...
How can you determine if someone is a successful clinician educator? Is there a certain level of success (or area of mastery) that needs to be achieved?
Do markers of success/mastery differ among institutions? Among departments/ units?  Are any markers constant?
Do mentees know what level of mastery is expected of them?
Do you think your mentee’s estimations of their level of mastery are aligned with yours?
Is there ever a point in the mentoring relationship in which the mentee is so independent that they no longer need the mentor?

## Slide 6
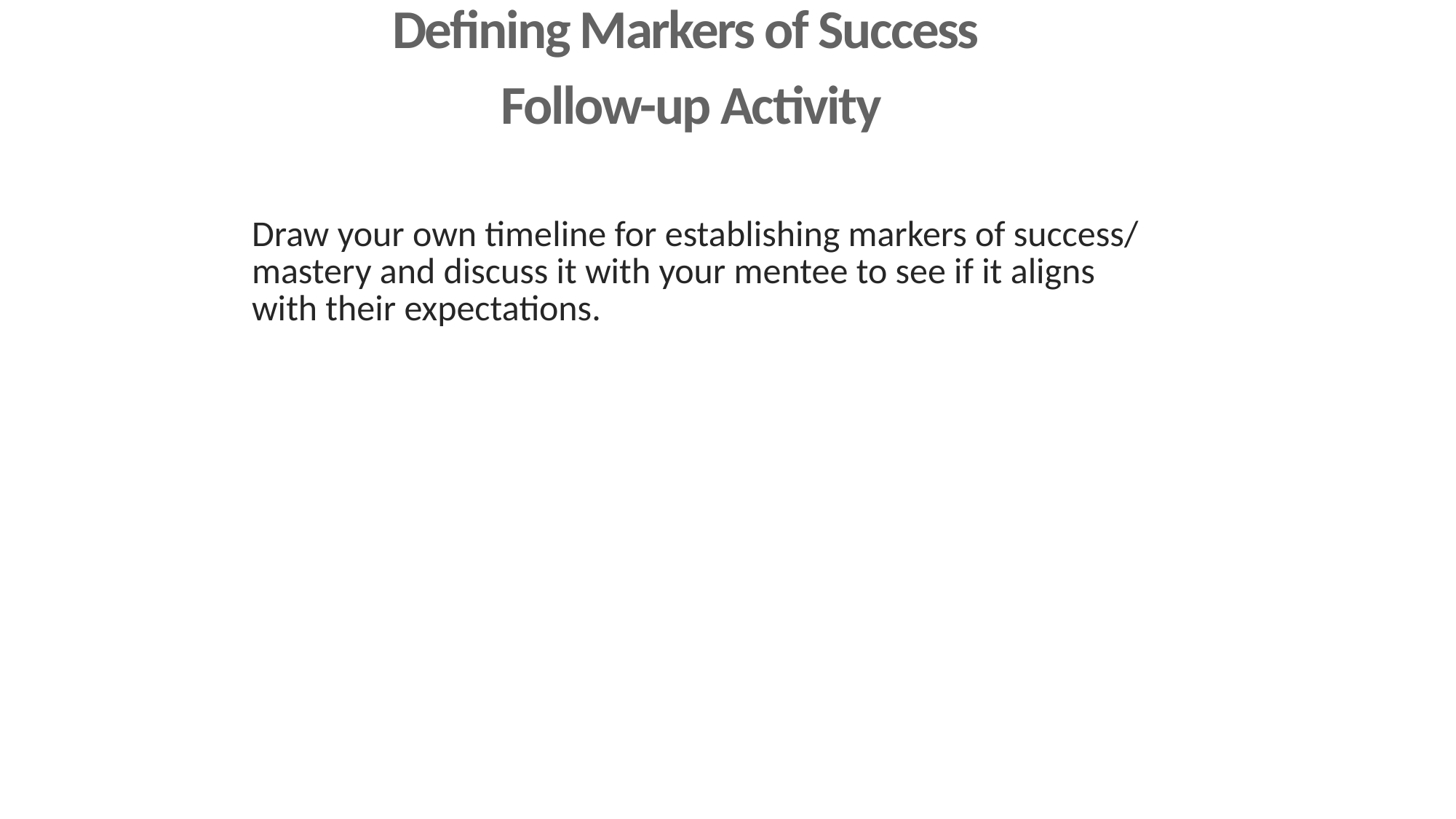

# Defining Markers of Success
Follow-up Activity
Draw your own timeline for establishing markers of success/ mastery and discuss it with your mentee to see if it aligns with their expectations.

## Slide 7
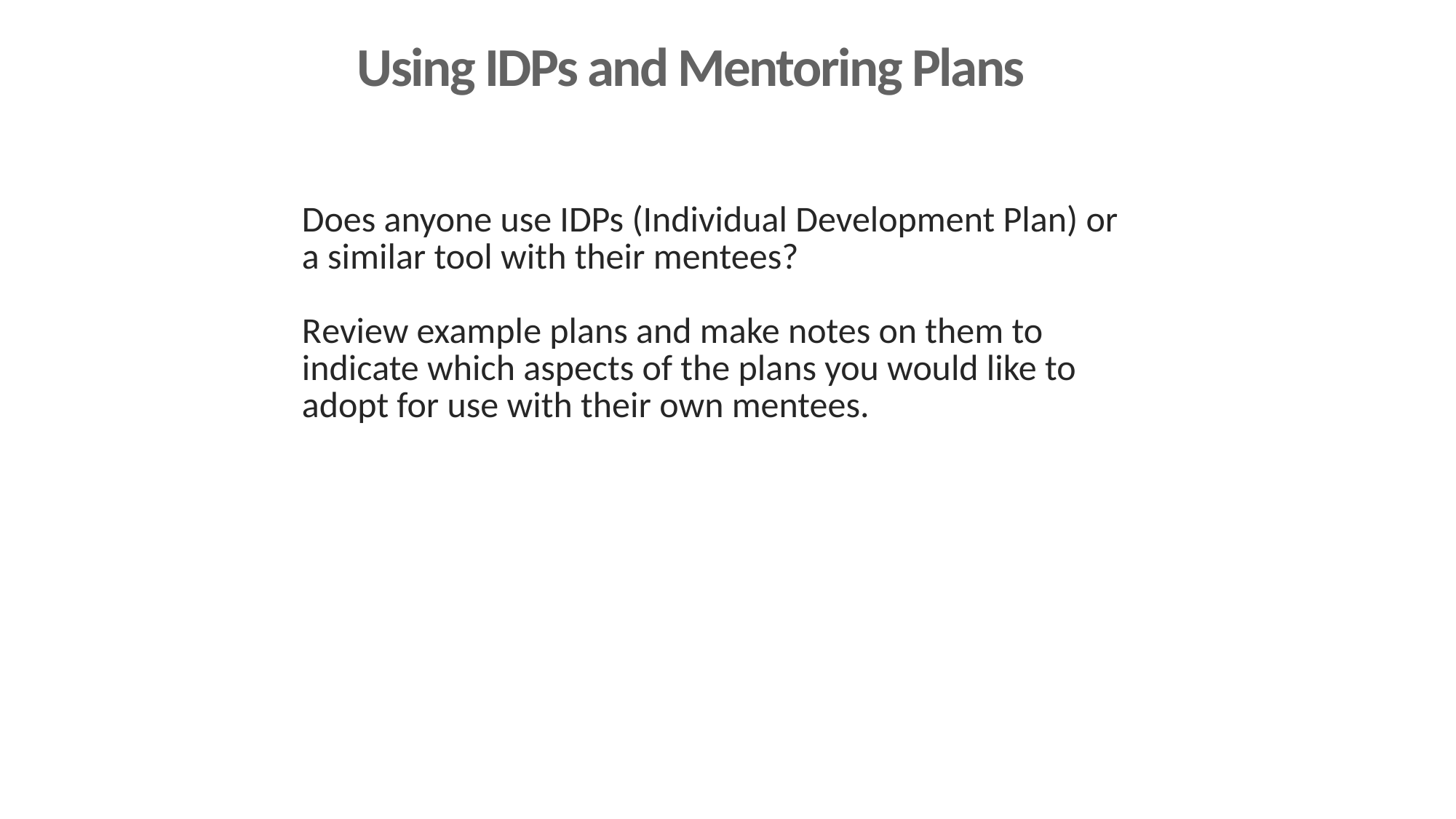

# Using IDPs and Mentoring Plans
Does anyone use IDPs (Individual Development Plan) or a similar tool with their mentees?
Review example plans and make notes on them to indicate which aspects of the plans you would like to adopt for use with their own mentees.

## Slide 8
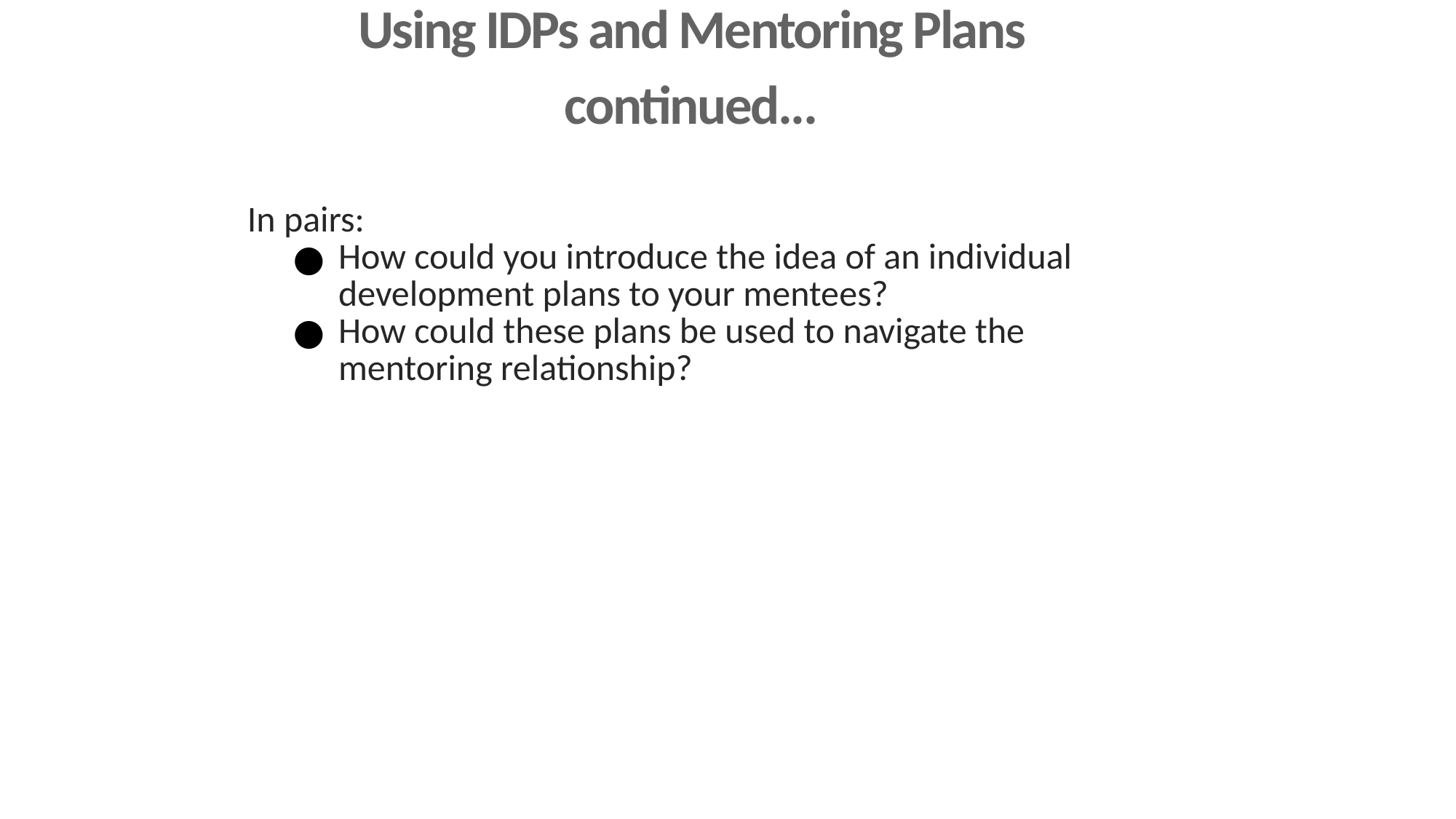

# Using IDPs and Mentoring Plans continued...
In pairs:
How could you introduce the idea of an individual development plans to your mentees?
How could these plans be used to navigate the mentoring relationship?

## Slide 9
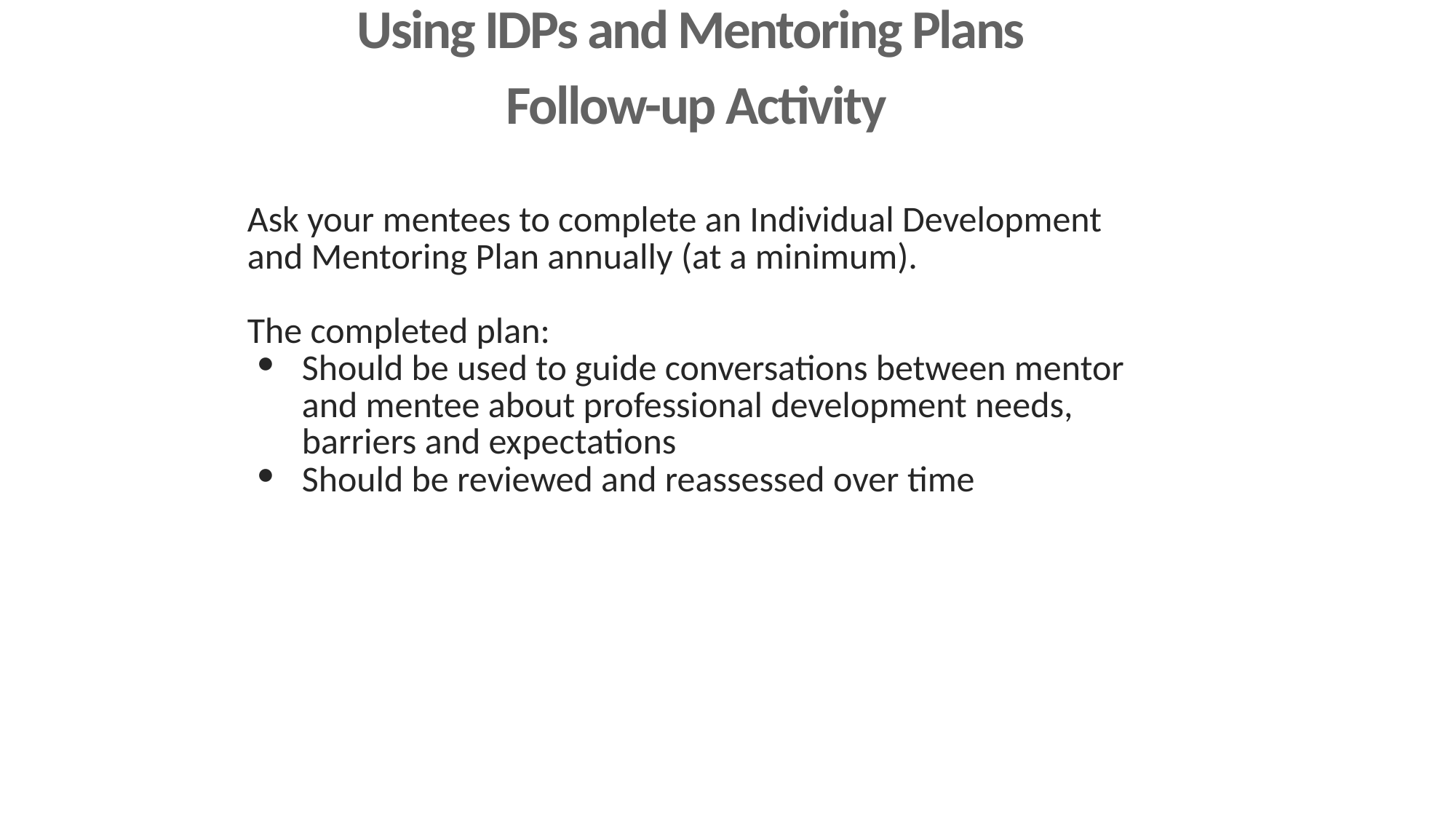

# Using IDPs and Mentoring Plans Follow-up Activity
Ask your mentees to complete an Individual Development and Mentoring Plan annually (at a minimum).
The completed plan:
Should be used to guide conversations between mentor and mentee about professional development needs, barriers and expectations
Should be reviewed and reassessed over time

## Slide 10
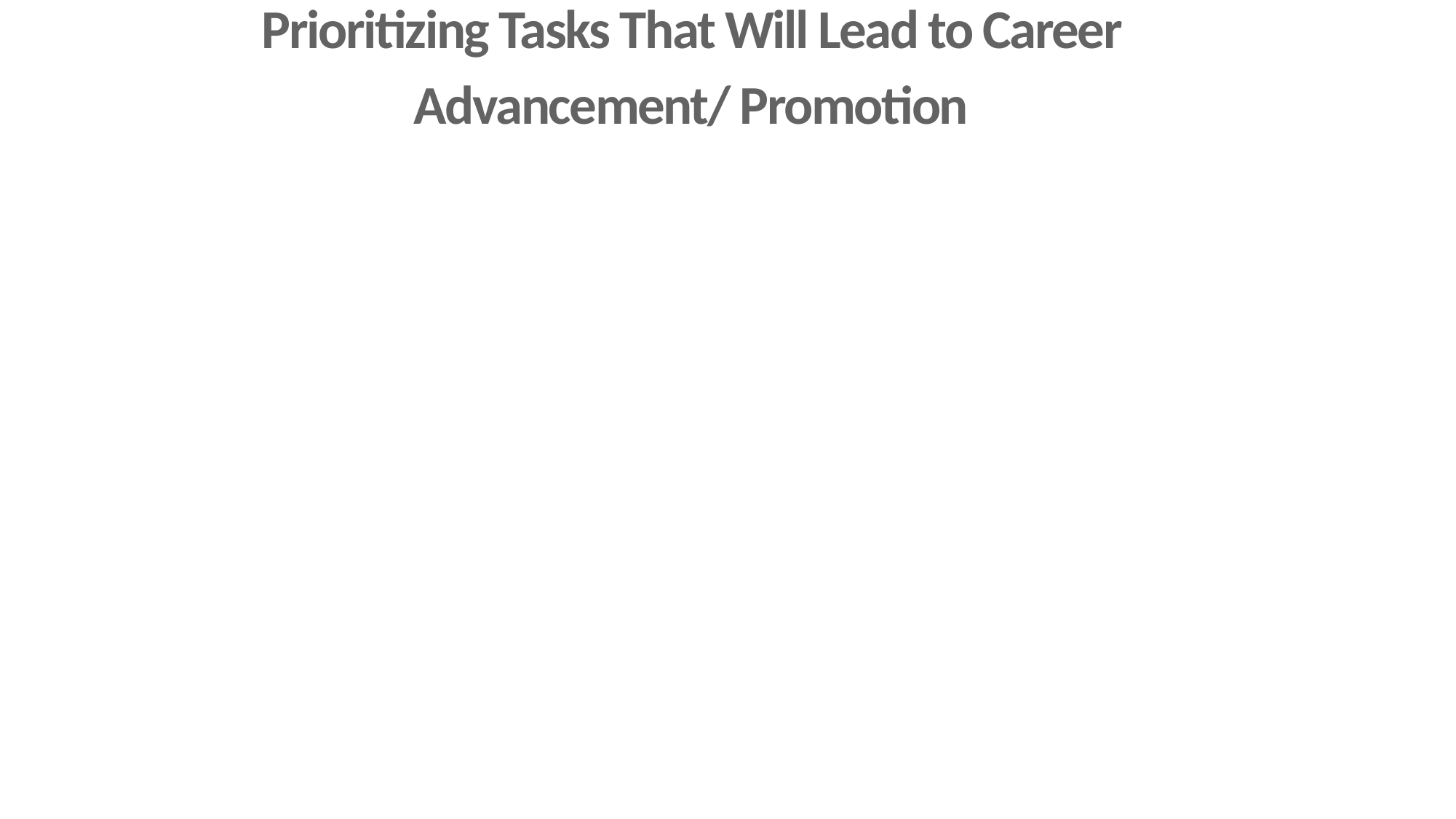

# Prioritizing Tasks That Will Lead to Career Advancement/ Promotion

## Slide 11
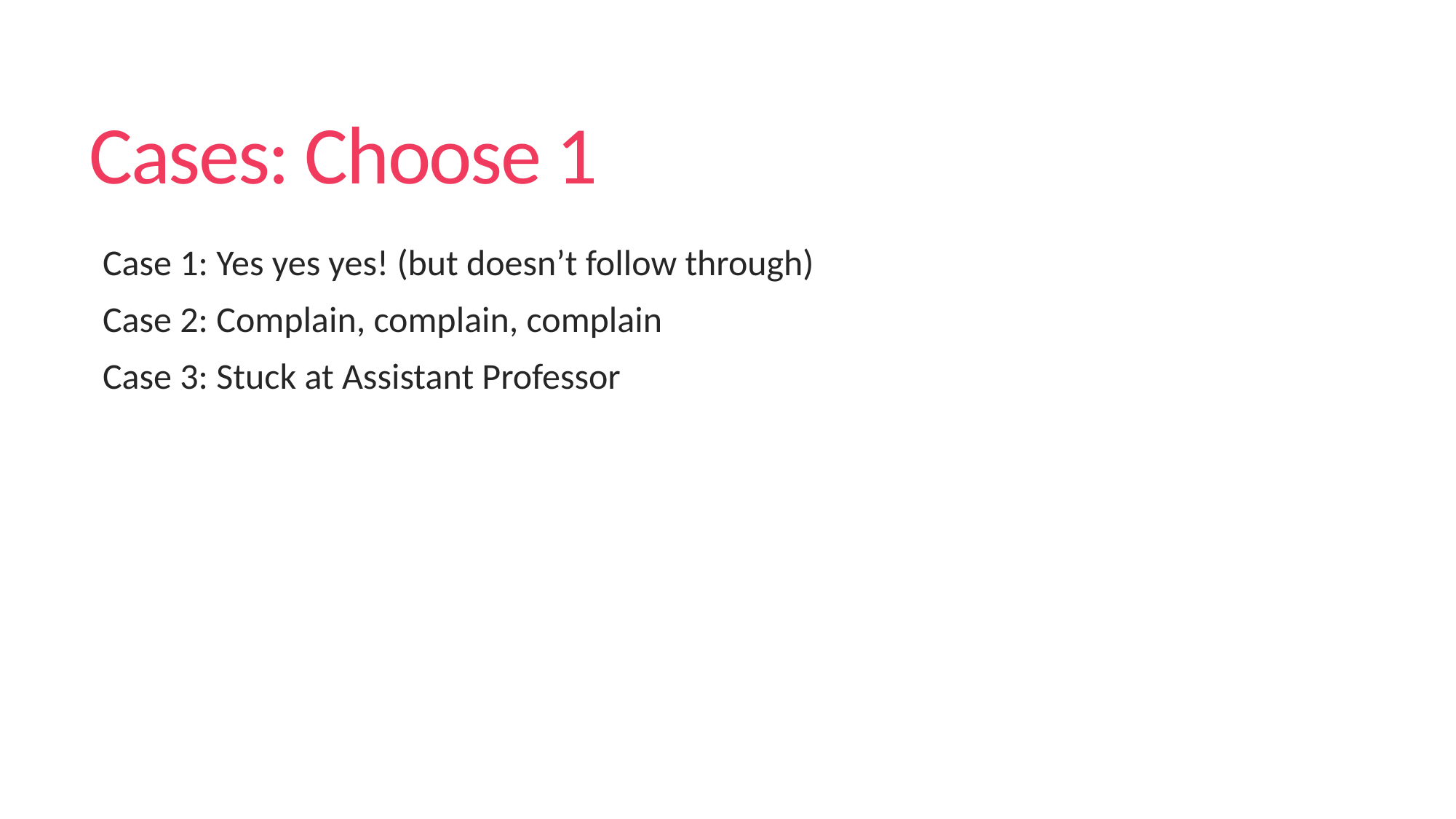

# Cases: Choose 1
Case 1: Yes yes yes! (but doesn’t follow through)
Case 2: Complain, complain, complain
Case 3: Stuck at Assistant Professor

## Slide 12
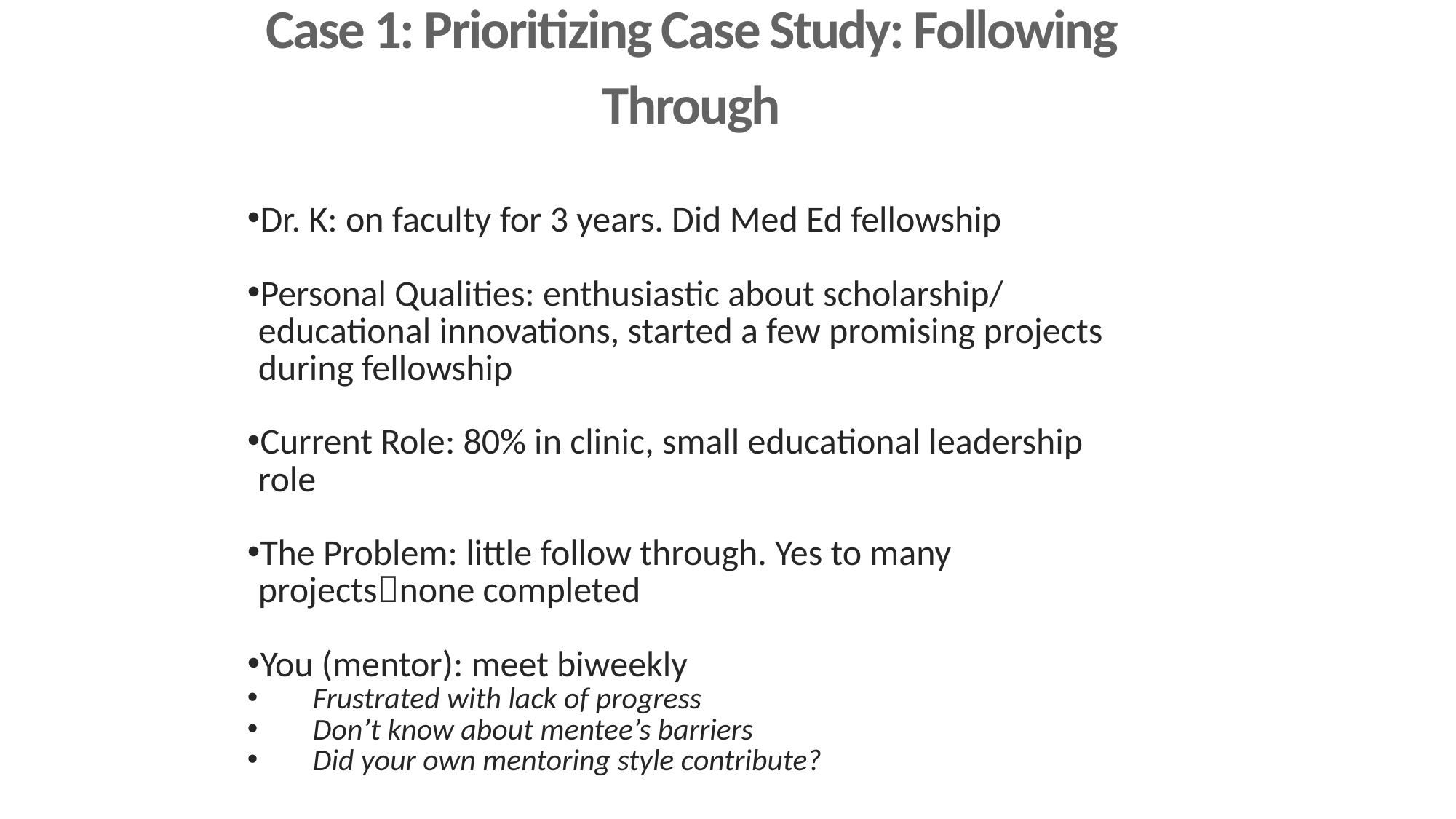

# Case 1: Prioritizing Case Study: Following Through
Dr. K: on faculty for 3 years. Did Med Ed fellowship
Personal Qualities: enthusiastic about scholarship/ educational innovations, started a few promising projects during fellowship
Current Role: 80% in clinic, small educational leadership role
The Problem: little follow through. Yes to many projectsnone completed
You (mentor): meet biweekly
Frustrated with lack of progress
Don’t know about mentee’s barriers
Did your own mentoring style contribute?

## Slide 13
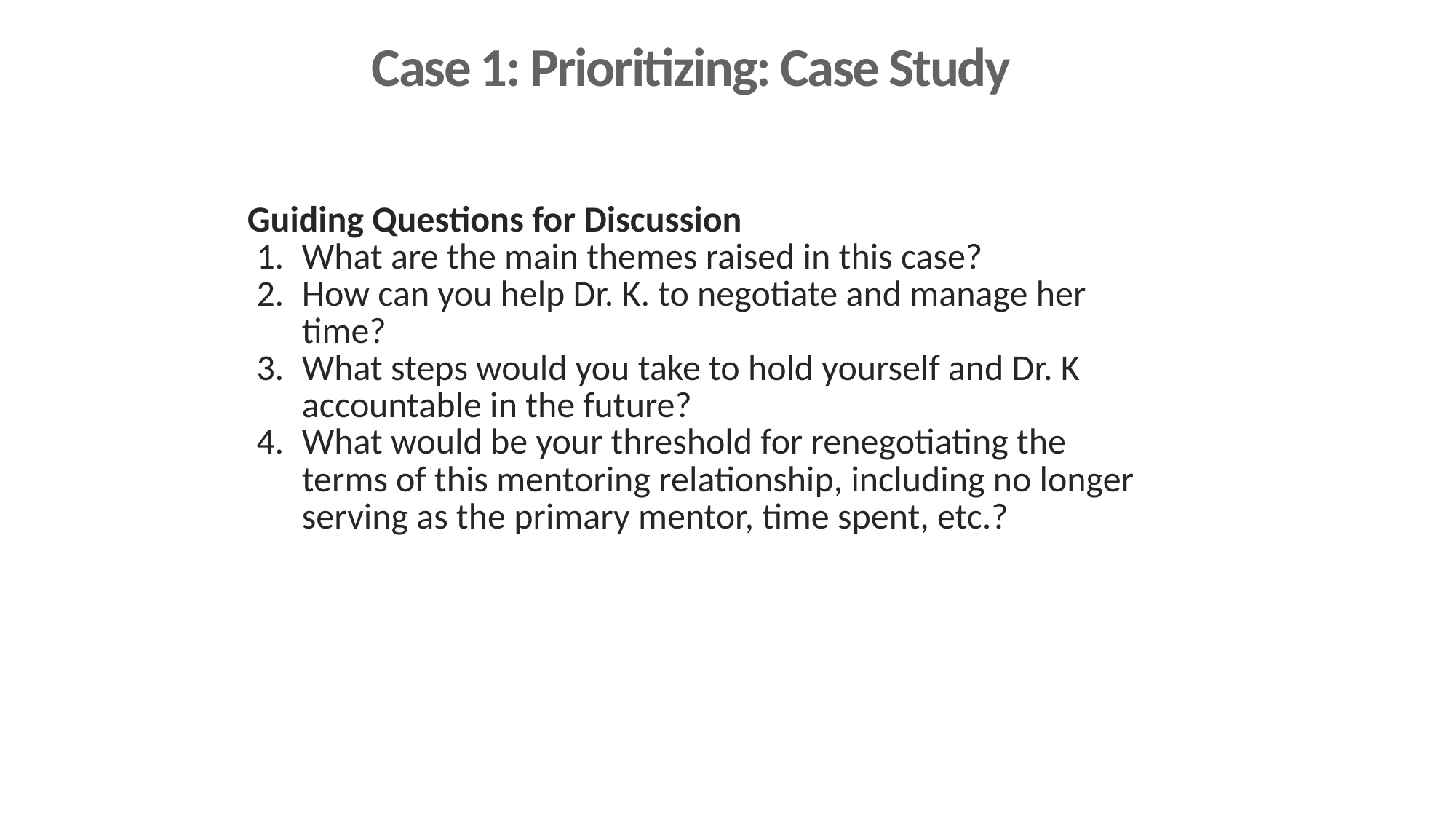

# Case 1: Prioritizing: Case Study
Guiding Questions for Discussion
What are the main themes raised in this case?
How can you help Dr. K. to negotiate and manage her time?
What steps would you take to hold yourself and Dr. K accountable in the future?
What would be your threshold for renegotiating the terms of this mentoring relationship, including no longer serving as the primary mentor, time spent, etc.?

## Slide 14
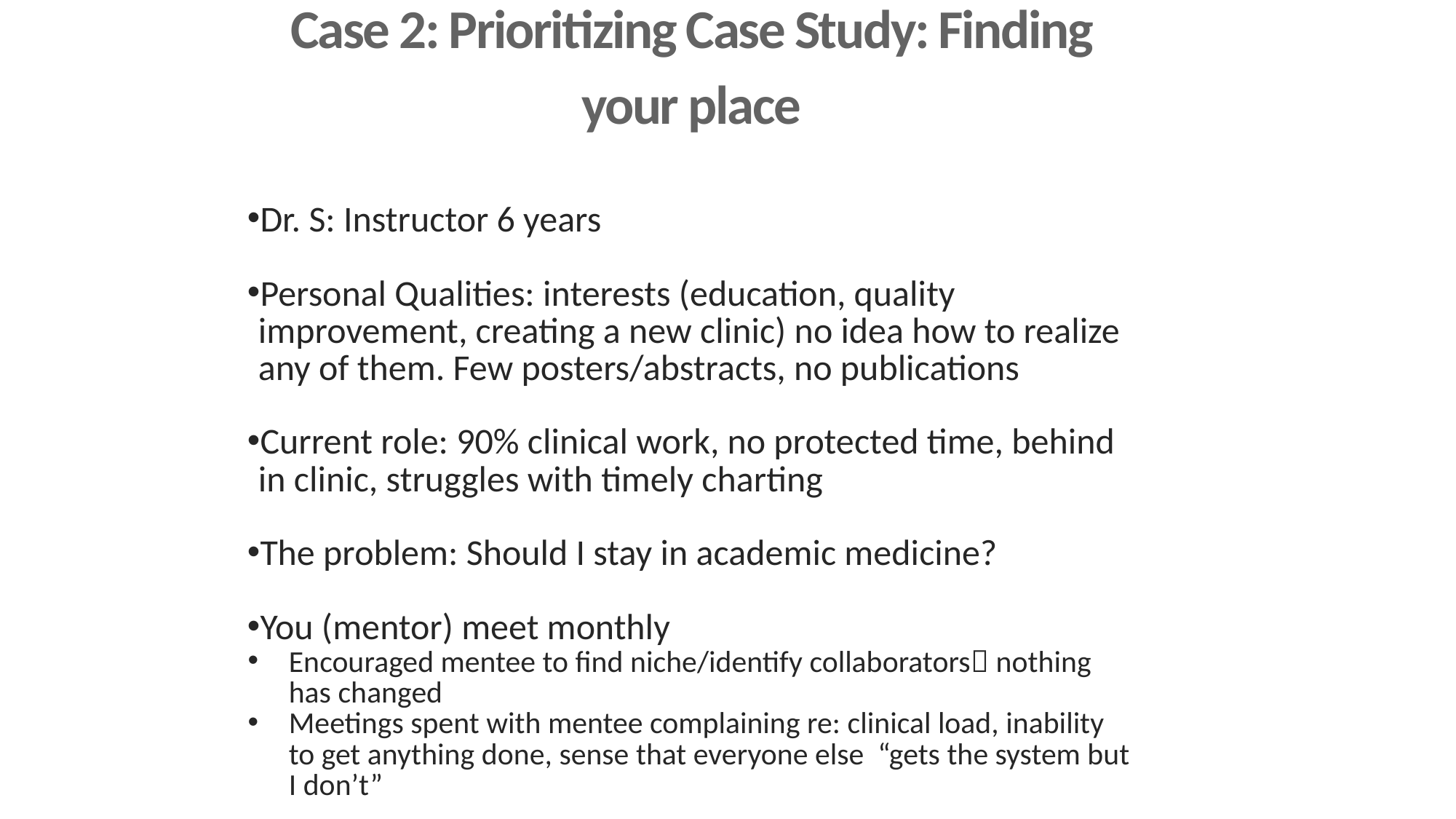

# Case 2: Prioritizing Case Study: Finding your place
Dr. S: Instructor 6 years
Personal Qualities: interests (education, quality improvement, creating a new clinic) no idea how to realize any of them. Few posters/abstracts, no publications
Current role: 90% clinical work, no protected time, behind in clinic, struggles with timely charting
The problem: Should I stay in academic medicine?
You (mentor) meet monthly
Encouraged mentee to find niche/identify collaborators nothing has changed
Meetings spent with mentee complaining re: clinical load, inability to get anything done, sense that everyone else “gets the system but I don’t”

## Slide 15
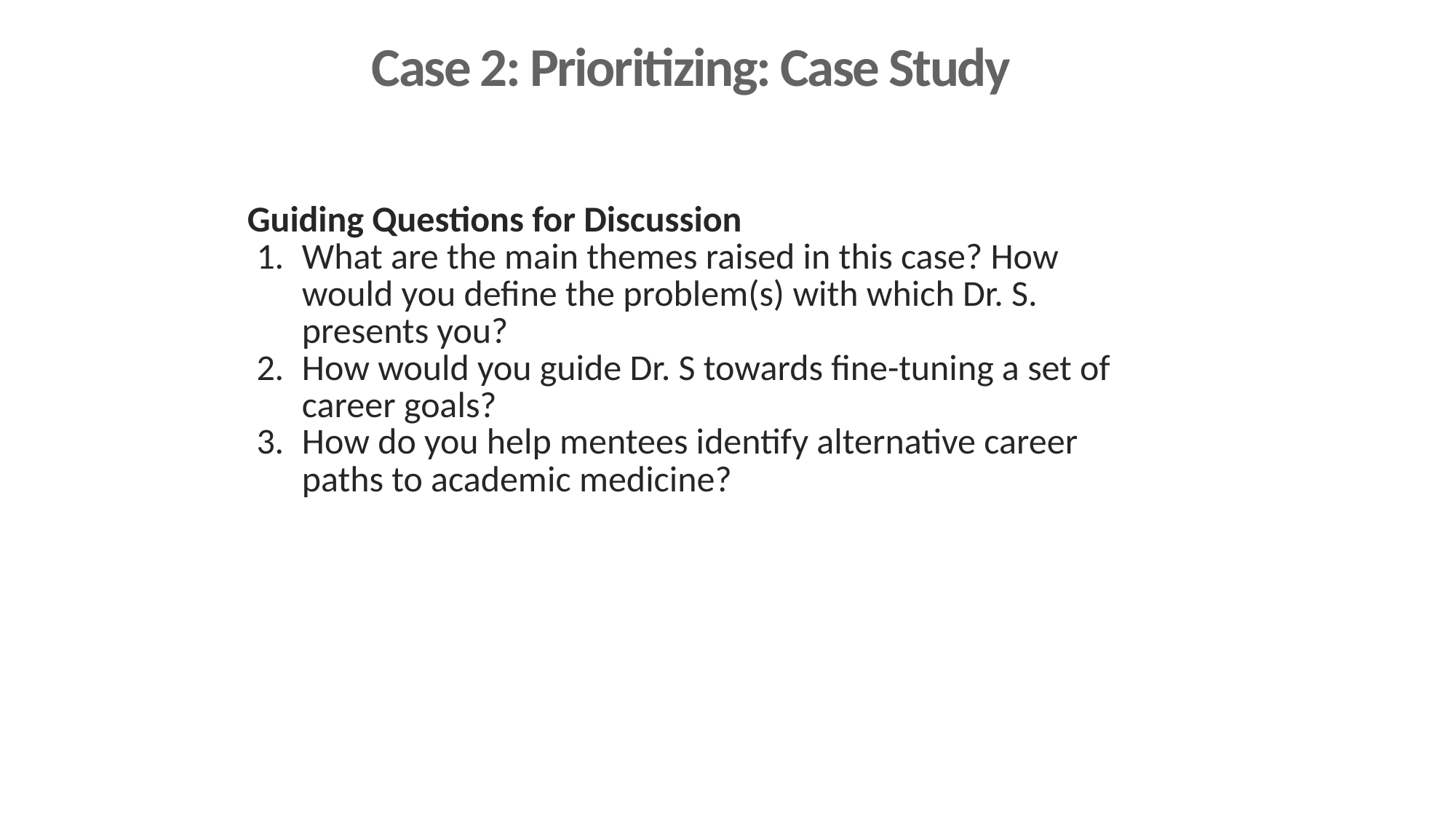

# Case 2: Prioritizing: Case Study
Guiding Questions for Discussion
What are the main themes raised in this case? How would you define the problem(s) with which Dr. S. presents you?
How would you guide Dr. S towards fine-tuning a set of career goals?
How do you help mentees identify alternative career paths to academic medicine?

## Slide 16
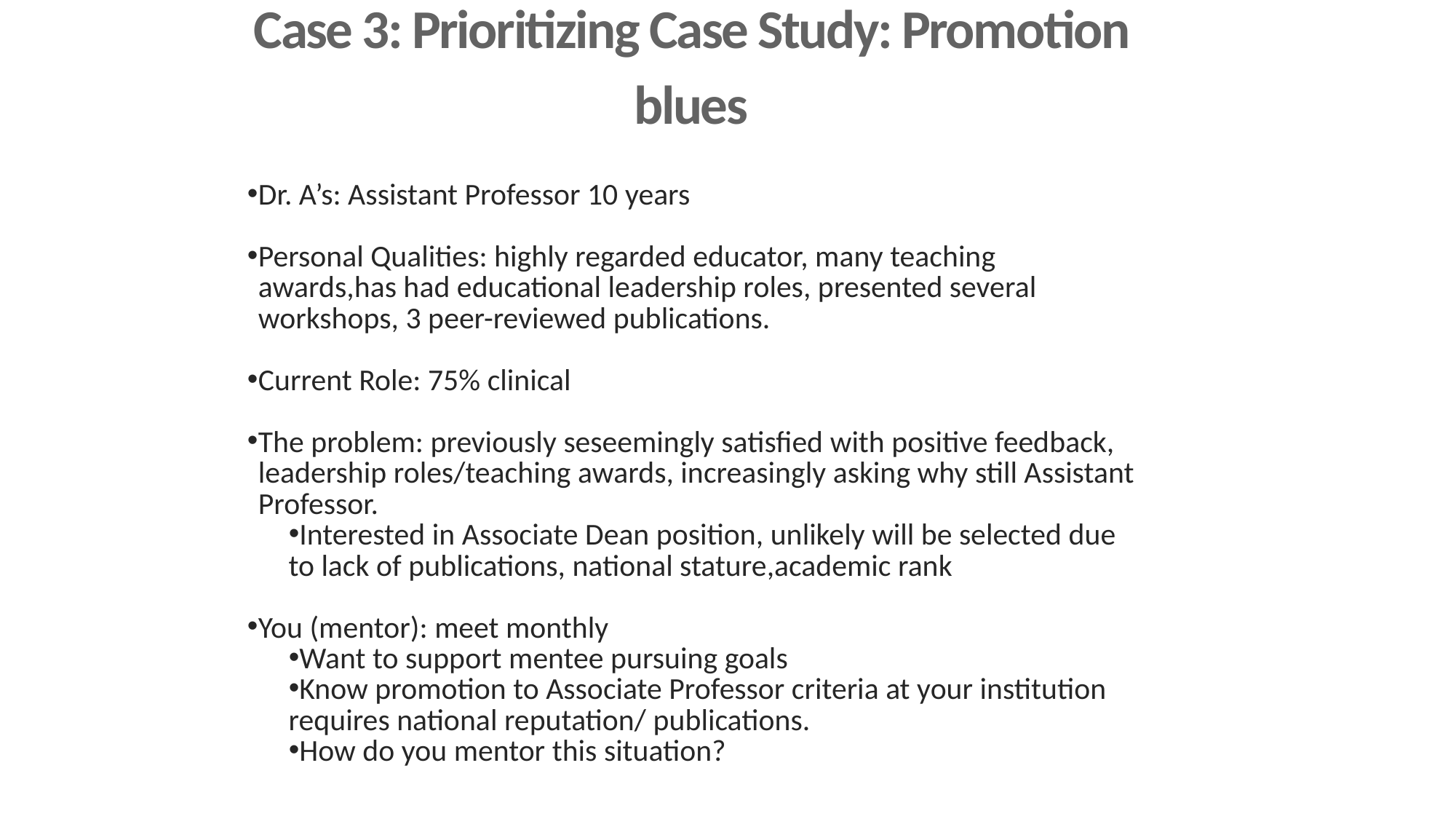

# Case 3: Prioritizing Case Study: Promotion blues
Dr. A’s: Assistant Professor 10 years
Personal Qualities: highly regarded educator, many teaching awards,has had educational leadership roles, presented several workshops, 3 peer-reviewed publications.
Current Role: 75% clinical
The problem: previously seseemingly satisfied with positive feedback, leadership roles/teaching awards, increasingly asking why still Assistant Professor.
Interested in Associate Dean position, unlikely will be selected due to lack of publications, national stature,academic rank
You (mentor): meet monthly
Want to support mentee pursuing goals
Know promotion to Associate Professor criteria at your institution requires national reputation/ publications.
How do you mentor this situation?

## Slide 17
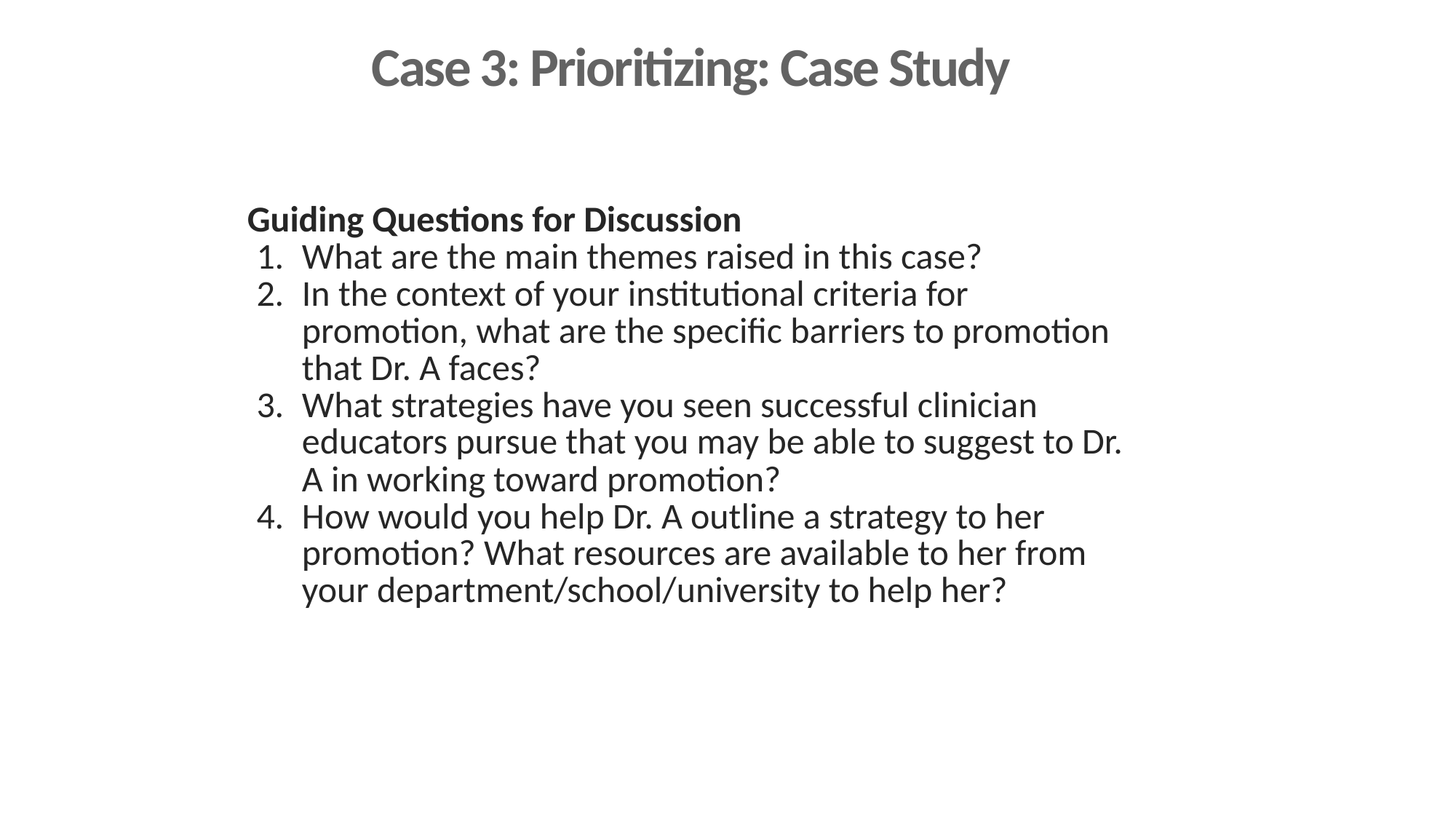

# Case 3: Prioritizing: Case Study
Guiding Questions for Discussion
What are the main themes raised in this case?
In the context of your institutional criteria for promotion, what are the specific barriers to promotion that Dr. A faces?
What strategies have you seen successful clinician educators pursue that you may be able to suggest to Dr. A in working toward promotion?
How would you help Dr. A outline a strategy to her promotion? What resources are available to her from your department/school/university to help her?

## Slide 18
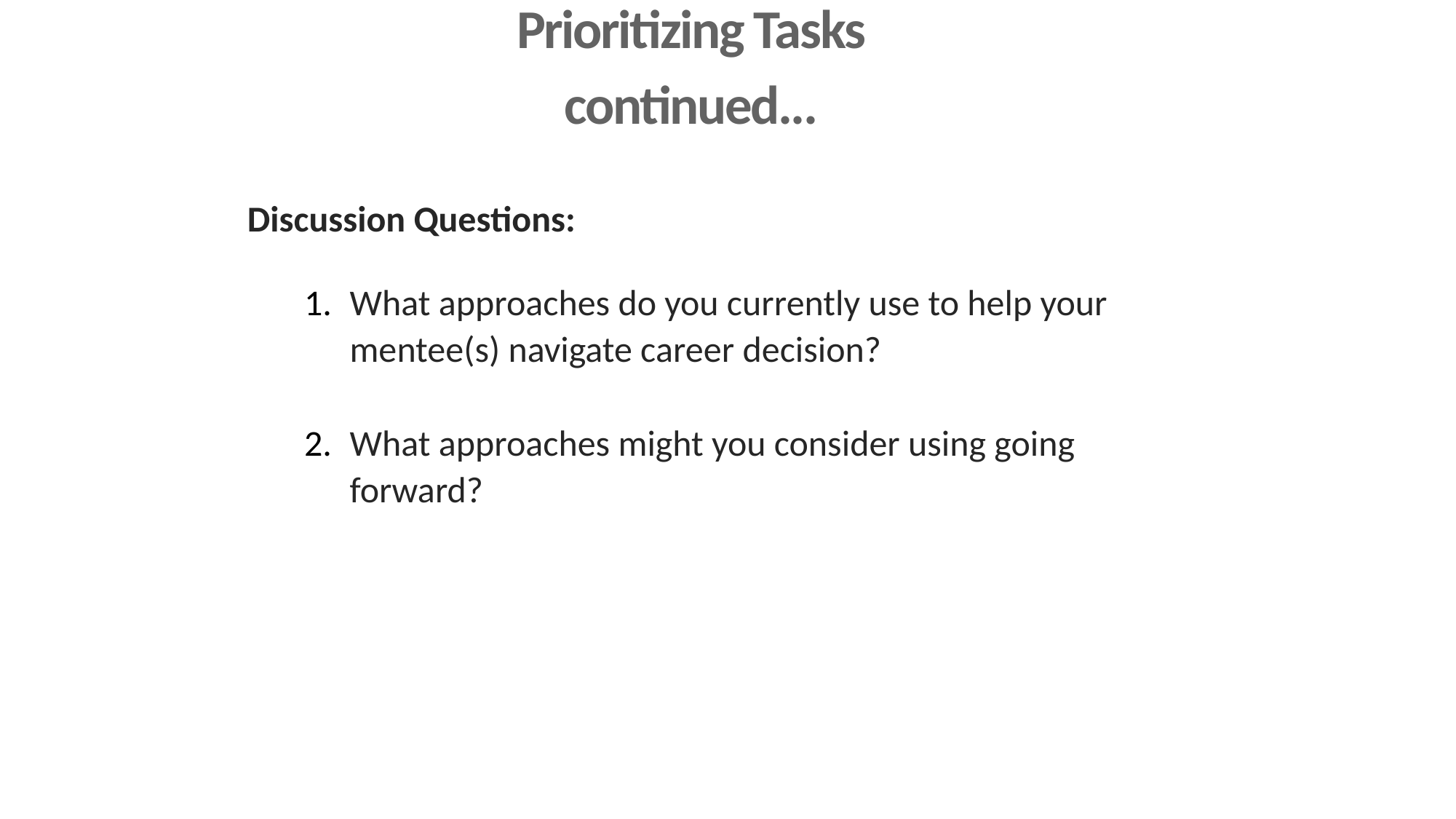

# Prioritizing Tasks
continued...
Discussion Questions:
What approaches do you currently use to help your mentee(s) navigate career decision?
What approaches might you consider using going forward?

## Slide 19
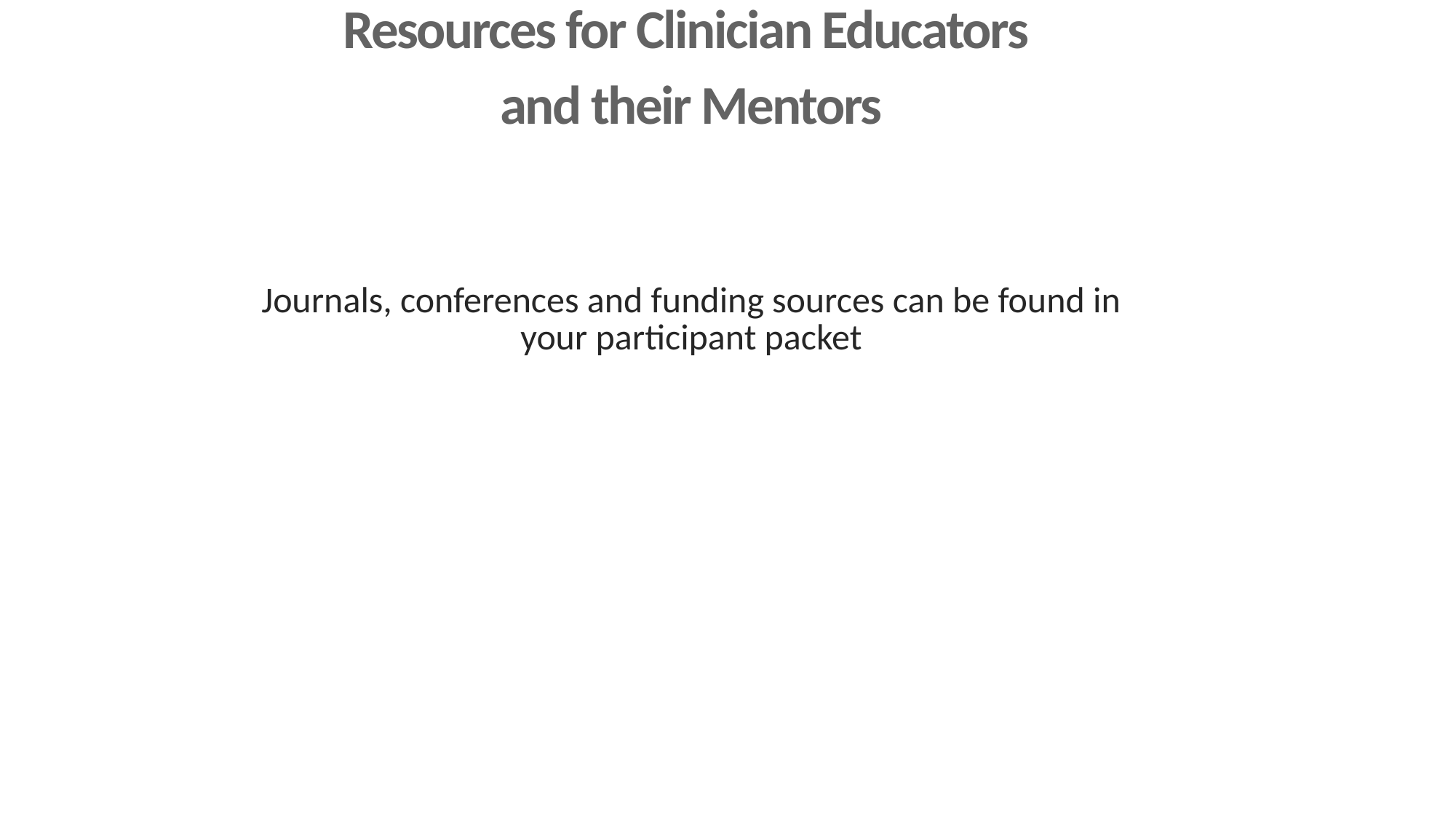

# Resources for Clinician Educators
and their Mentors
Journals, conferences and funding sources can be found in your participant packet

## Slide 20
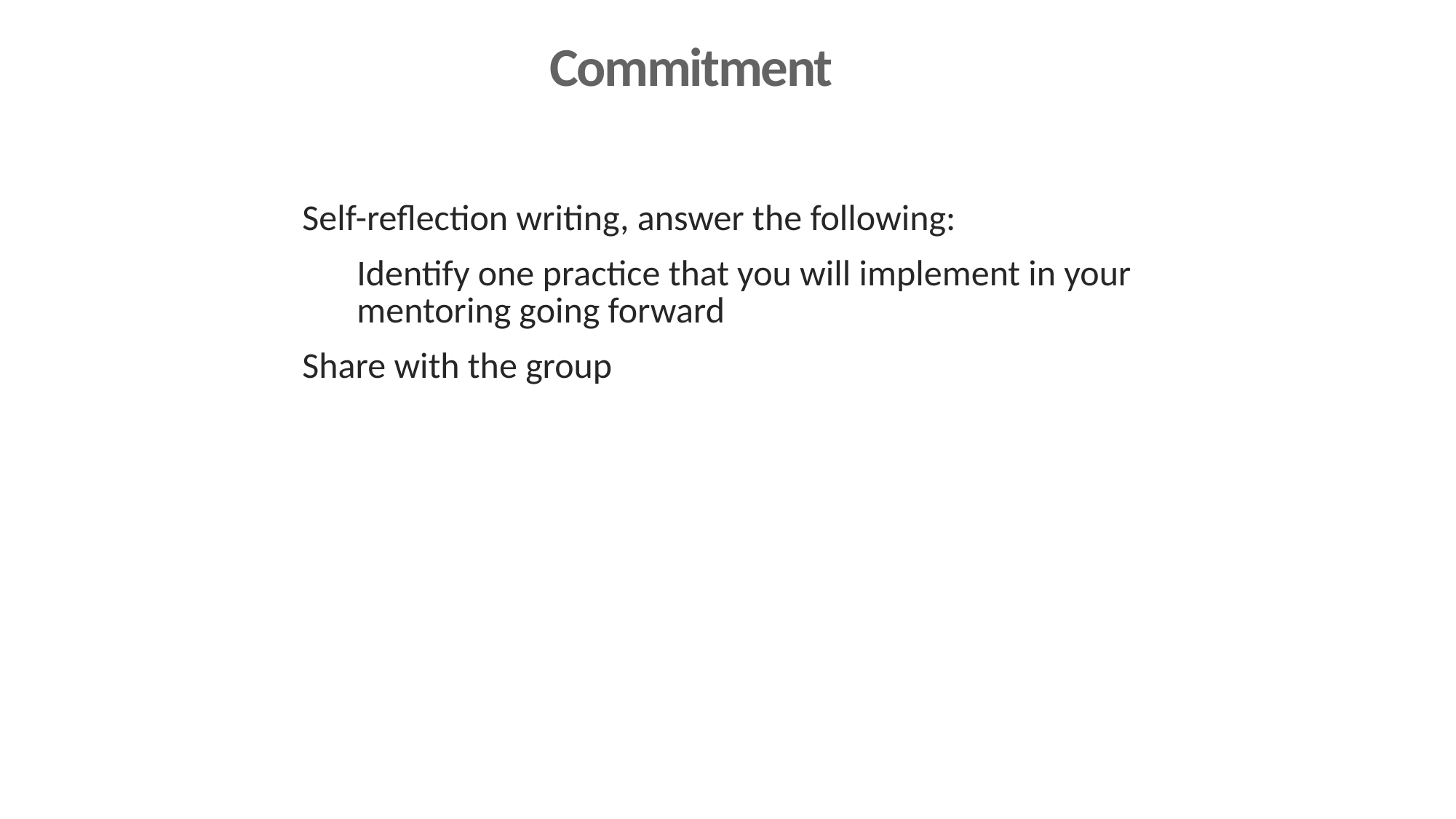

# Commitment
Self-reflection writing, answer the following:
Identify one practice that you will implement in your mentoring going forward
Share with the group

## Slide 21
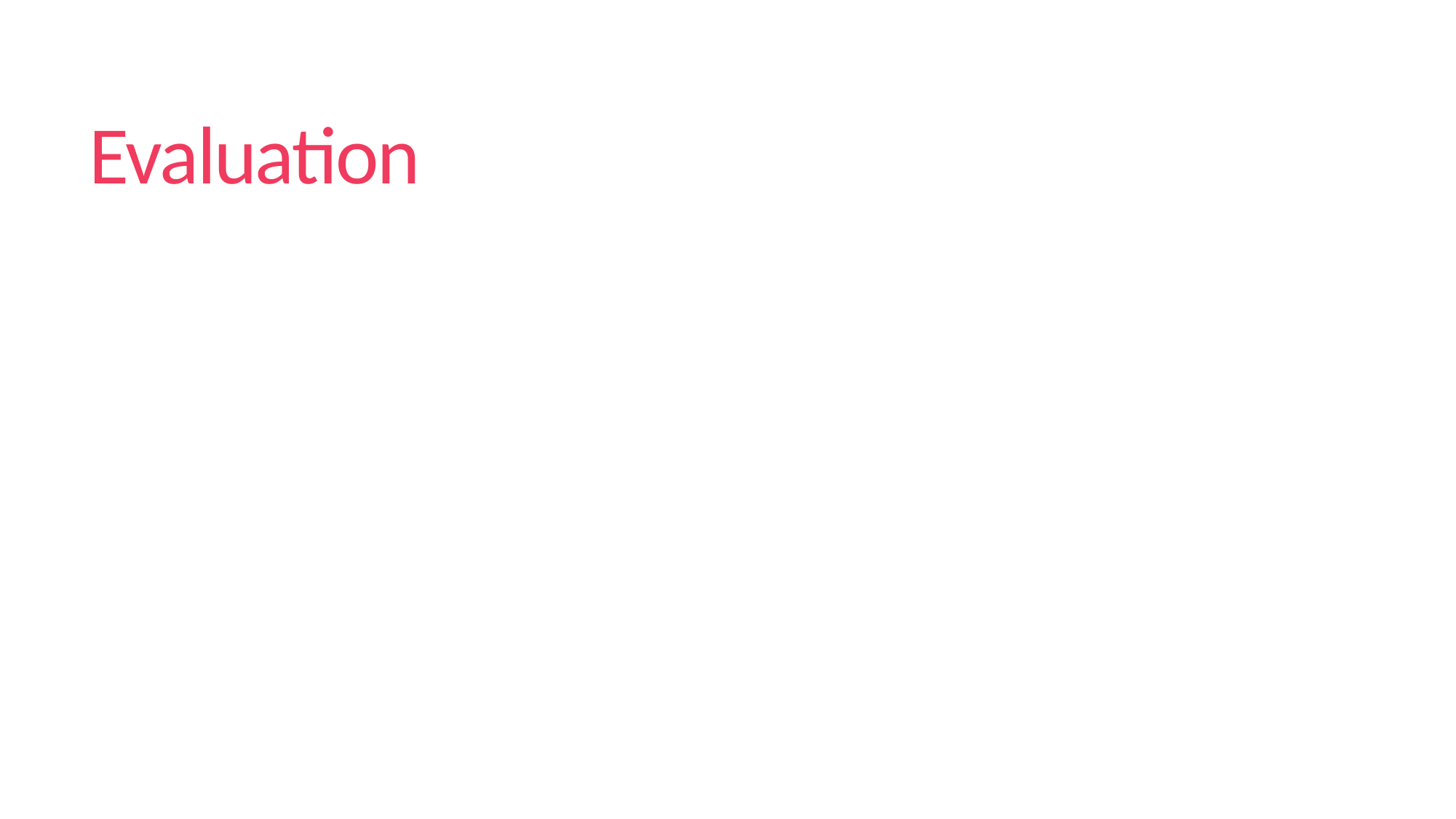

# Evaluation

## Slide 22
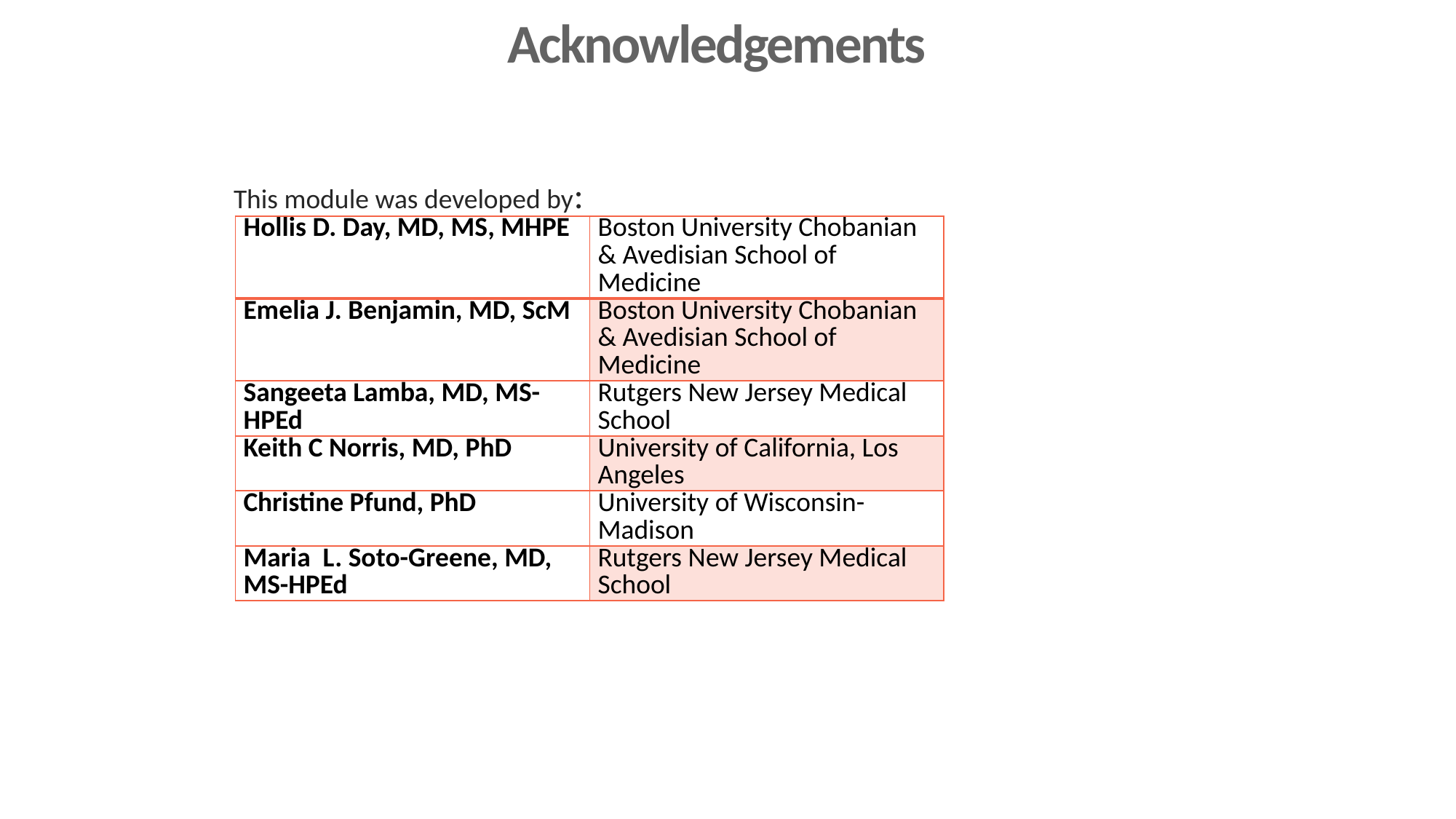

# Acknowledgements
This module was developed by:
| Hollis D. Day, MD, MS, MHPE | Boston University Chobanian & Avedisian School of Medicine |
| --- | --- |
| Emelia J. Benjamin, MD, ScM | Boston University Chobanian & Avedisian School of Medicine |
| Sangeeta Lamba, MD, MS-HPEd | Rutgers New Jersey Medical School |
| Keith C Norris, MD, PhD | University of California, Los Angeles |
| Christine Pfund, PhD | University of Wisconsin-Madison |
| Maria L. Soto-Greene, MD, MS-HPEd | Rutgers New Jersey Medical School |

## Slide 23
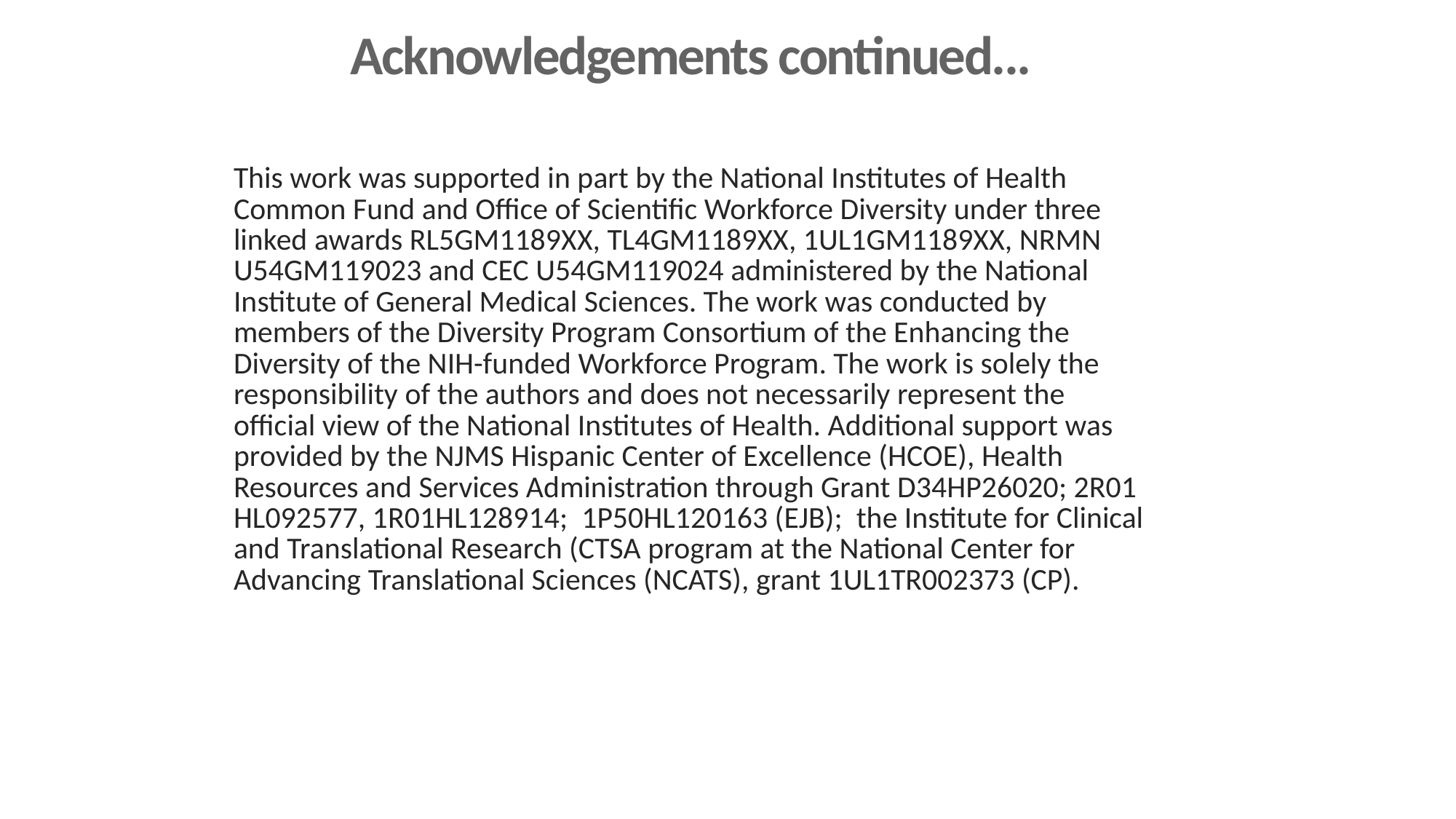

# Acknowledgements continued...
This work was supported in part by the National Institutes of Health Common Fund and Office of Scientific Workforce Diversity under three linked awards RL5GM1189XX, TL4GM1189XX, 1UL1GM1189XX, NRMN U54GM119023 and CEC U54GM119024 administered by the National Institute of General Medical Sciences. The work was conducted by members of the Diversity Program Consortium of the Enhancing the Diversity of the NIH-funded Workforce Program. The work is solely the responsibility of the authors and does not necessarily represent the official view of the National Institutes of Health. Additional support was provided by the NJMS Hispanic Center of Excellence (HCOE), Health Resources and Services Administration through Grant D34HP26020; 2R01 HL092577, 1R01HL128914; 1P50HL120163 (EJB); the Institute for Clinical and Translational Research (CTSA program at the National Center for Advancing Translational Sciences (NCATS), grant 1UL1TR002373 (CP).
