# Supplementary material for: Fostering Success and Promoting Professional Development of Clinician Educator Mentees: A Workshop for Mentors
Source: MedEdPORTAL. 2023 Jun 27;19:11321. doi: 10.15766/mep_2374-8265.11321 (PMC10293477; doi:10.15766/mep_2374-8265.11321)
Supplement: Supplementary file 1 — CE Training Workshop.pptxFacilitator Guide.docxIndividual Development and Mentoring Plans.docxCase Studies.docxResource Guide.docxWorkshop Evaluation.docx [file mep_2374-8265.11321-s001.zip › C. Individual Development and Mentoring Plans.docx]

***Sample Individual Development Plans*****

***Example #1: Individual Development Plan (IDP)***

**1. Name**_____________________________________

**2. Date______________________**

**3. Academic Series and Rank**

| Ladder Rank  In-Residence  Adjunct  Clinical  Health Science Clinical | Instructor  Assistant  Associate  Professor |
| --- | --- |

**4. Primary Mentor_____________________________________________** Additional Mentor(s)**__________________________________________**

**5. Identify Personal and Institutional Long Term Goals**

*Why did you decide to work at a medical school?*

*What do you personally hope to accomplish in your career?*

*List your Academic Series requirements (see Academic Criteria for Series)*

List other goals discussed with Chair/Division head

**6. Areas of Focus: Definition and Distribution of Effort**

The following six areas of focus generally describe the areas in which faculty direct their efforts to successfully accomplish their personal, institutional, and academic series goals.

**• Teaching—Excellence in Education**

Teaching, student advising, continuing medical education (CME), new course development

**• Research/Creative Activity—Leadership in Innovative Educational Research**

Conducting educational research, presentations, publications, application for and receipt of grant support, editing, and peer review

**• Clinical Care—State-of-the-Art Clinical Care**

Direct patient care, chart review, related clinical activities, and clinical budget performance.

Clinical scholarship may include co-authoring case reports, clinical guidelines and other

**• Service—Leadership in Governance**

Participation or leadership in governance, committee membership, collegial activities

Suggested service priority: Department, SOM, UCDHS, University, professional community

**• Self Development—Networking, Work-Life Integration, and Additional Mentors**

Faculty Development activities, leadership programs, CME training, earning advanced degrees, participation in professional academic associations or societies, developing professional contacts, consulting in one’s field, expanding network contacts, balancing work and personal life, utilizing additional mentors in specific areas of focus

**Distribution of Effort**

Estimate the hours per week spent in each focus area, then list the percentage of total duties.

| **Focus Area** | **# Hrs/Week** | **% of Total Duties** |
| --- | --- | --- |
| **Teaching** |  |  |
| **Scholarship** |  |  |
| **Clinical Care** |  |  |
| **Community Engagement/Service** |  |  |
| **Administration/Institutional Service** |  |  |
| **Self-Development**  **(Networking, Work-Life Balance, Additional Mentors)** |  |  |
| **Total** |  |  |

**7. Specific Goals in Focus Areas**

Complete the focus areas that specifically apply to the criteria for your academic success, and will help you accomplish your personal and institutional long- term goals.

**Teaching**

Year in Review: Please list last year’s goal(s) and significant accomplishments (teaching appointments, invitations, course or program improvements, etc).

If the goals were not met, explain, and identify barriers.

*Upcoming year’s teaching goal(s):*

*Identify resources, collaborators, and time commitment needed to achieve goal(s):*

*Identify barriers to achieving new goal(s):*

**Scholarship Related/Creative Activities**

Year in Review: Please list last year’s goal(s) and significant accomplishments (major publications, grants, presentations, invitations, etc). If the goals were not met, explain and identify barriers.

*Identify in a single sentence the focus of your scholarly activity.*

*Upcoming year’s research goal(s):*

*Identify resources, collaborators, and time commitment needed to achieve goal(s):*

*Identify barriers to achieving new goal(s):*

**Clinical Care**

Year in Review: Please list last year’s goal(s) and significant accomplishments (exceptional patient care, development of new techniques, clinical programs, etc). If the goals were not met, explain and identify barriers.

*Upcoming year’s patient care goal(s):*

*Identify resources, collaborators, and time commitment needed to achieve goal:*

*Identify barriers to achieving new goals:*

**Service**

Recommended service priority: Department, School, University, Professional, and Community.

Year in Review: Please list last year’s goal(s) and significant accomplishments. If the goals were not met, explain and identify barriers.

*Upcoming year’s administration goal(s):*

*Identify resources, collaborators, and time commitment needed to achieve goal:*

*Identify barriers to achieving new goal(s):*

**Self-Development (Networking, Work-Life Balance, Additional Mentors)**

Year in Review: Please list year’s goal(s) and significant accomplishments. If the goal were not met, explain and identify barriers.

*Upcoming year’s self-development goal(s):*

*Identify resources, collaborators, and time commitment needed to achieve goal(s):*

*Identify barriers to achieving new goal(s):*

**8. Optimal Distribution of Effort**

Revisit the table, “Distribution of Effort,” in step 6. Create a new Optimal Distribution of Effort table, taking into account your specific goals listed in step 7.

| **Focus Area** | **# Hours/Week** | **% of Total Duties** |
| --- | --- | --- |
| **Teaching** |  |  |
| **Research** |  |  |
| **Clinical Care** |  |  |
| **Community Engagement** |  |  |
| **Administration/Service** |  |  |
| **Self-Development**  **(Networking, Work/Life Balance and Additional Mentors)** |  |  |
| **Total** |  |  |

**9. We have met and discussed this annual Individual Development Plan (IDP)**

**Mentee ________________________________________________Date____________________**

**Mentor__**_______________________________________________**Date_**___________________

*****Adapted from IDP form from UC Davis Health System Accessed 8/15/2022 at: <https://health.ucdavis.edu/ctsc/area/education/mentoring-academy/PDFs/IDP_Template%20_%20General.pdf> (optional content)

***EXAMPLE #2: MENTORING PLAN WORKSHEET****

**Your Goals**

Prior to meeting with your mentor, take some time to think about and write down your clinical, education, research, and professional goals. You may want to articulate one- and five-year goals. For example, a short-term goal might be “to expand the patient referral base or develop a new course” and a long-term goal might be “to become a regional referral site for a particular procedure or present abstracts and publish the curriculum on MedEdPORTAL in order to gain regional recognition to meet promotion guidelines to Associate Professor.”

| **Short-term Goals (next year)** | **Long-term Goals (next 5 years)** |
| --- | --- |
| 1. | 1. |
| 2. | 2. |
| 3. | 3. |

**Potential Mentors**

Identify people who can assist you in meeting your goals. These can be mentors internally or at other institutions. Consider having a mentor in multiple domains, clinical, educational, scholarship, or service. For each potential mentor, identify objectives, develop a list of what you can offer, and propose outcomes.

A blank grid is included on the next page to help you organize your thoughts. Put your initial thoughts down on paper before you approach a mentor, and then revise it as your relationship changes.

**Approaching Mentors**

We suggest that you first approach mentors by sending an e-mail that includes a request for a meeting, your CV or Biosketch, a brief summary of your goals, and why you think there would be a good fit between you and the mentor. Let potential mentors know how you are hoping to work with them, such as one-on-one, as one of many mentors, or as part of a mentoring team or committee. You might want to let them know how you think they would be able to contribute.

**Identify Mentorship needs**

Identify competencies in which you will need to gain expertise (see Table below for examples). Identify people who can assist you in achieving these competencies and in meeting your goals. These can be mentors internally at your institution, or at other institutions. Put your initial thoughts down on paper before you approach a mentor, and then revise it as your relationship changes.

| Documenting clinical work | Establishing goals |
| --- | --- |
| Writing and performing QA/QI projects | Designing curriculum for educational projects |
| Managing your career | Managing staff |
| Leading teams | Preparing for promotion |
| Cultural humility | Navigating institution |
| Fostering diverse & inclusive culture, unconscious bias, having conversations about differences | Rigorous educational/program evaluation |
| Managing care | Managing conflict |
| Speaking before groups | Knowing career paths |
| Teaching effectively | Hiring personnel |
| Collaborating effectively | Managing budgets |
| Managing and analyzing data | Mentoring others |
| Giving and receiving feedback | Evaluating literature |
| Assessing students | Medical informatics |
| Organizational dynamics | Documenting institutional engagement |
| Developing experiential workshops | Networking at national meetings |
| Scholarly writing | Designing curriculum for MedEd Portal |

**Managing Relationships with Your Mentors**

Relationships should be nurtured and respected. If you and your proposed mentor develop a working relationship, have some guidelines for how you will work together. Here are some tips:

- Schedule standing meetings ahead of time, keep them, be punctual, and be prepared
- Send an agenda the day before the meeting
- Give your mentor(s) plenty of time to review drafts of projects; warn your mentor if you have a time sensitive request (e.g. abstract deadline)
- Don’t be a black hole of need – limit the number of requests you make of any given mentor
- Develop publication timelines and plans so that expectations are clear
- Clarify what your mentor’s preferred modes for scheduling meetings, communication, timelines, etc.
- Saying thank you is priceless

| **Mentoring Plan** | | | |
| --- | --- | --- | --- |
| ***Mentor Name*** | ***Objectives***  *(e.g., understand how*  *to develop scholarship in education)* | ***What I can offer***  *( e.g. writing abstracts, presentations, publications)* | ***Outcomes***  *(e.g. submit*  *curriculum or results in a peer reviewed format)* |
|  |  |  |  |
|  |  |  |  |

*Adapted from Ann J Brown, MD MHS, Vice Dean for Faculty, Duke University School of Medicine

**University of Massachusetts Individual Opportunity Plan***

**Faculty Name: Date:**

**STEP 1. Setting Your Career Mission**

What do you want to achieve? Where do you want to go? How do you want to make a difference?

**My career mission statement** *(be succinct)***:**

**STEP 2. Self-Assessment: Personal SWOT Analysis**

**My Strengths:**

What **skills** do you do well? What are the **strengths** in your knowledge base? What positive **behaviors** or **attributes** do you exhibit? *(Highlight strengths that are relevant to your mission statement)*

1.

2.

3.

4.

5.

**My Weaknesses:**

What **skills**, **behaviors** or **attributes** do you wish to acquire or improve? What are the gaps in your **knowledge**? What **resources** or **connections** are you missing? *(Highlight weaknesses that are relevant to your mission statement)*

1.

2.

3.

4.

5.

**Opportunities:**

Where is the **growth** opportunity for you? What is **changing** in your field? What **funding** opportunities are available? What are the **gaps in knowledge**? Look for opportunities, openings, changes in your department, school, community, nationally…

1.

2.

3.

4.

**Threats:**

What **threats** (**barriers**, **obstacles**) are present or predicted in your department, the school or the external world that might hinder you accomplishing your mission? *What are the* ***political*** *minefields?* *Changes in* ***funding****?*

1.

2.

3.

4.

5.

**STEP 3. Planning Your Career**

How will you achieve your career mission? What are your goals and immediate objectives?

**My career goals for the next 3–5 years:***(In order of priority. Indicate when you expect to complete the goal.)*

1.

2.

3.

4.

**Objectives for the next year:**

*Write an objective for a goal that you plan to advance in the next year.
Use the S.M.A.R.T. approach to write your objectives.
(Duplicate this page for each of your objectives.)*

**Goal:**

**Objective:**

*What personal* ***strengths*** *does this objective build on?*

*What personal* ***weaknesses*** *are addressed by this objective?*

*What* ***opportunities*** *will you seize to achieve this goal?*

*How will you address* ***threats*** *to achieving this goal?*

*What* ***resources*** *do you need to achieve this objective:
time, money, space, expertise (mentoring), personnel, equipment, materials?*

*Adapted from UMass Chan Medical School Office of Faculty Affairs Individual Opportunity Plan worksheet Accessed 8/15/2022 from <https://www.umassmed.edu/ofa/development/iop/step1/> (optional content)

** Additional sample IDPs are available at: https://mentoringresources.ictr.wisc.edu. Mentors may also refer their mentees to <http://myidp.sciencecareers.org>. This was initially designed for PhD faculty but the website offers a guided, online process to develop a plan. (optional content)
