# Supplementary material for: Fostering Success and Promoting Professional Development of Clinician Educator Mentees: A Workshop for Mentors
Source: MedEdPORTAL. 2023 Jun 27;19:11321. doi: 10.15766/mep_2374-8265.11321 (PMC10293477; doi:10.15766/mep_2374-8265.11321)
Supplement: Supplementary file 1 — CE Training Workshop.pptxFacilitator Guide.docxIndividual Development and Mentoring Plans.docxCase Studies.docxResource Guide.docxWorkshop Evaluation.docx [file mep_2374-8265.11321-s001.zip › D. Case Studies.docx]

**CASE STUDIES**

**Case 1: Following through**

You are a mid-career faculty member and primary mentor for Dr. K, an early career faculty member who is three years out of the medical education fellowship at your institution. Dr. K shows a lot of potential to have a successful clinician educator career. This mentee is bright, passionate about medical education, and enthusiastic to launch an academic career.

Dr. K spends 80% of time seeing patients in clinic and has taken on a small educational leadership role in the department. Dr. K is committed to developing educational innovations and scholarship, and has started a few promising projects during fellowship.

Dr. K always seems to have good intentions but there has been little follow through. Dr. K enthusiastically says yes to many projects. Despite bi-weekly mentor meetings, this mentee continues to have uneven progress, with no projects brought to fruition. You aren’t sure how to talk to Dr. K about your concerns: on the one hand you are frustrated with the lack of progress and follow through despite having spent lots of time meeting. On the other hand, you don’t know what barriers Dr. K is facing and how your own mentoring style may have contributed to Dr. K’s failure to meet milestones.

***Guiding Questions for Discussion***

1. What are the main themes raised in this case?
2. How can you help this mentee to negotiate and manage their time?
3. What steps would you take to hold yourself and Dr. K accountable in the future?
4. What would be your threshold for renegotiating the terms of this mentoring relationship, including no longer serving as the primary mentor, time spent, etc.?

**Case 2**: **Finding your place**

Dr. S is an early career clinician educator who has recently come to you for career mentoring. Dr. S has many widespread interests from education to quality improvement to creating a new clinic but has no idea how to begin or how to realize any of them. Dr. S is still an Instructor, despite having been on faculty for several years. Dr. S has presented a few posters and abstracts but has not published any peer-reviewed papers.

Since none of these interests are funded, Dr. S does a lot of clinical work. Though well-liked by colleagues and patients, Dr. S is often behind in clinic, and struggles to keep chart completion up to date. Dr. S does not have any protected time during the workweek to pursue other interests. This mentee is beginning to question whether academic medicine is the right career choice.

As a mentor, you have encouraged Dr. S to find a niche, and to identify other faculty members who may have similar interests to collaborate with, but nothing has changed. Increasingly, your meetings are spent with the mentee complaining about the growing frustrations with the clinical load, the inability to get anything else done, and the sense that every other colleague seems to understand “the system” when the mentee doesn’t.

***Guiding Questions for Discussion***

1. What are the main themes raised in this case? How would you define the problem(s) with which Dr. S. presents you?
2. How would you guide Dr. S towards fine-tuning a set of career goals?
3. How do you help mentees identify alternative career paths to academic medicine?

**Case 3**: **Promotion blues**

You have been one of Dr. A’s mentors for many years. Dr. A is a highly regarded educator at your institution, who has been recognized for teaching excellence by students and faculty. Dr. A has consistently had outstanding teaching evaluations, and has served in departmental educational leadership roles, but has remained at the rank of Assistant Professor for the past ten years. Dr. A has attended annual medical education conferences and presented several educational workshops and has three peer-reviewed publications.

It has always seemed like Dr. A has been satisfied by feeling recognized by students and colleagues through positive feedback, leadership roles, and teaching awards, but increasingly this mentee has been asking about remaining at the rank of Assistant Professor.  The mentee is interested in pursuing a position as an Associate Dean, but you are aware that it is unlikely that Dr. will be selected because of the lack of publications, national stature, and academic rank.

You think highly of Dr. A and want to support any academic goals, but are aware that the criteria for promotion to Associate Professor at your institution require faculty to have a national reputation and more peer reviewed publications. You are unsure how to effectively mentor Dr. A.

***Guiding Questions for Discussion***

1. What are the main themes raised in this case?
2. In the context of your institutional criteria for promotion, what are the specific barriers to promotion that Dr. A faces?
3. What strategies have you seen successful clinician educators pursue that you may be able to suggest to Dr. A in working toward promotion?
4. How would you help Dr. A outline a strategy for promotion? What resources are available from your department/school/university to assist the process?
