# Supplementary material for: Fostering Success and Promoting Professional Development of Clinician Educator Mentees: A Workshop for Mentors
Source: MedEdPORTAL. 2023 Jun 27;19:11321. doi: 10.15766/mep_2374-8265.11321 (PMC10293477; doi:10.15766/mep_2374-8265.11321)
Supplement: Supplementary file 1 — CE Training Workshop.pptxFacilitator Guide.docxIndividual Development and Mentoring Plans.docxCase Studies.docxResource Guide.docxWorkshop Evaluation.docx [file mep_2374-8265.11321-s001.zip › E. Resource Guide.docx]

**Resources for Clinician Educators and their Mentors**

**Journals for Educational Scholarship**

- To help clinician educators identify journals to publish in, the AAMC-Regional Groups on Educational Affairs Medical Education Scholarship, Research and Evaluation Section created an ***Annotated Bibliography of Journals for Educational Scholarship***, which is posted at the website: <https://www.aamc.org/members/gea/>. The current version is at <https://www.aamc.org/system/files/2019-11/prodev-affinity-groups-gea-annotated-bibliography-journal-educational-scholarship-110619.pdf>
- To publish health professions curricula, and teaching and assessment resources, consider submitting to **MedEdPORTAL**, a peer-reviewed open access online journal of the AAMC - <https://www.mededportal.org/>
- To identify the journals that are the best fit for your manuscript, consider using the Journal/Author Name Estimator (**JANE**): <http://jane.biosemantics.org/>

**Conferences for Clinical Educators**

- **AMEE** Annual International Conference for medical and healthcare professions education - <https://amee.org/conferences>
- International Association of Medical Science Educators (**IAMSE**) Annual Meeting for those who teach and lead curricula in sciences of medicine and health – [iamse.org](http://www.cvent.com/events/22nd-annual-iamse-meeting/event-summary-6ee327232ea047e9bbfed93c39692c19.aspx)
- Association of Standardized Patient Educators (**ASPE**) Annual Conference for human simulation education - <http://www.aspeducators.org/future-conferences>
- Team Based Learning Collaborative (**TBLC**) Annual Conference for educators of all disciplines - <http://www.teambasedlearning.org/>
- The Ottawa Conference on the Assessment of Competence in Medicine and the Healthcare Professions & International Conference on Medical Education (**ICME**) Conference for health professions educators - https://www.ottawaconference.org/
- Collaborating Across Borders, an interprofessional education conference held alternately in the US and Canada- https://aihc-us.org/activities/cab
- All Together Better Health, an international conference that focuses on interprofessional education - https://atbh.org/
- Curated list of medical education conferences by **MedEd World**: A Global Medical Education Community - <https://www.mededworld.org/Conferences/Upcoming-Conferences.aspx>

**Funding Sources for Medical Education Research**

- Agency for Healthcare Research and Quality (AHRQ) - <https://www.ahrq.gov/funding/index.html>
- Alfred P. Sloan Foundation - <https://sloan.org/grants/apply/>
- AMEE- <https://amee.org/AMEE/Grants/AMEE/Grants_Landing.aspx?hkey=c06f81f8-279b-4dc5-9b1d-2dc404a3647f>
- American Board of Internal Medicine (ABIM) Foundation - <http://abimfoundation.org/what-we-do/grants>
- Arnold P. Gold Foundation - <http://www.gold-foundation.org/programs/research/mtl/>
- The Association of American Medical Colleagues Group on Educational Affairs (GEA) is accepting research applications that foster collaborations among GEA sections or regions, or within an institution. <https://www.aamc.org/search?keys=mesre%20grant>
- Arthur Vining Davis Foundations - <http://www.avdf.org/Grants/GrantsOverview.aspx>
- Commonwealth Fund - <http://www.commonwealthfund.org/grants-and-fellowships/grants>
- Health Resources and Services Administration (HRSA) - <https://www.hrsa.gov/grants/index.html>
- Independent Medical Education - <https://www.bms.com/about-us/responsibility/IME.html>
- Josiah Macy Jr. Foundation - <http://www.macyfoundation.org/apply>
- National Board of Medical Examiners Stemmler Medical Education Research Fund - <http://www.nbme.org/research/stemmler.html>
- National Science Foundation - <https://www.nsf.gov/funding/index.jsp>
- Physicians Foundation - https://physiciansfoundation.org/grants/
- Society for Academic Continuing Medical Education (SACME) - <https://sacme.org/SACME_Grants/>
- Teleflex Medical Education Grants - <https://www.teleflex.com/usa/en/about-us/grants/medical-educational-grants/medical-educational-grant-request/>

Of note, various medical specialty societies also fund educational research, such as:

- Association of Professors of Gynecology and Obstetrics <https://apgo.org/page/grantsawards>

Biosketches are frequently required in both competing applications and progress reports. Find instructions, blank format pages, and sample biosketches on this NIH website: <https://grants.nih.gov/grants/forms/biosketch.htm>

**Selected References about Medical Education Research Grants and Funding**

- Gruppen LD and Durning SJ. Needles and Haystacks: Finding Funding for Medical Education Research. *Acad Med*. 2016;91(4):480-4.
- Blanco MA, Gruppen LD, Artino AR, Jr., Uijtdehaage S, Szauter K and Durning SJ. How to write an educational research grant: AMEE Guide No. 101. *Med Teach*. 2016;38(2):113-22.
- Experimental study design and grant writing in eight steps and 28 questions. Georges Bordage & Beth Dawson Medical Education 2003;37(4):376–385.

**Selected References on Medical Education Mentorship**

1. Atasoylu AA, Wright SM, Beasley BW, Cofrancesco J, Jret al. Promotion criteria for clinician-educators. *J Gen Intern Med*. 2003;18(9):711-6.

2. Bertram A, Yeh HC, Bass EB, Brancati F, et al. How we developed the GIM clinician-educator mentoring and scholarship program to assist faculty with promotion and scholarly work. *Med Teach*. 2015;37(2):131-5.

3. Blanchard RD, Nagler A, Artino AR, Jr. Harvest the Low-Hanging Fruit: Strategies for Submitting Educational Innovations for Publication. *J Grad Med Educ*. 2015;7(3):318-22.

4. Blanchard RD, Visintainer PF, La Rochelle J. Cultivating Medical Education Research Mentorship as a Pathway Towards High Quality Medical Education Research. *J Gen Intern Med*. 2015;30(9):1359-62.

5. Castiglioni A, Aagaard E, Spencer A, Nicholson L, Karani R, Bates CK, Willett LL and Chheda SG. Succeeding as a Clinician Educator: useful tips and resources. *J Gen Intern Med*. 2013;28(1):136-40.

6. Cristancho S, Varpio L. Twelve tips for early career medical educators. *Med Teach*. 2016;38(4):358-63.

7. Crites J, GainesK, Cottrell S, Kalishman S, et al. Medical education scholarship: An introductory guide: AMEE Guide No. 89. *Med Teach*. 2014; 36(8):657-74.

8. Bordages G. Considerations on preparing a paper for publication. *Teach Learn Med*. 1989; 1:1, 47-52.

9. Gillespie SM, Thornburg LL, Caprio TV, Medina-Walpole A. Love letters: an anthology of constructive relationship advice shared between junior mentees and their mentors. *J Grad Med Educ*. 2012;4(3):287-9.

10. Hu WC, Thistlethwaite JE, Weller J, Gallego G, et al. 'It was serendipity': a qualitative study of academic careers in medical education. *Med Educ*. 2015;49(11):1124-36.

11. Li ST, Gusic ME, Vinci RJ, Szilagyi PG, Klein MD. A structured framework and resources to use to get your medical education work published. *MedEdPORTAL*. 2018;14:10669.

12. Reader S, Fornari A, Simon S and Townsend J. Promoting Faculty Scholarship - An evaluation of a program for busy clinician-educators. *Can Med Educ J*. 2015;6:e43-60.

13. Simpson DE, Marcdante KW, Duthie EH Jr, Sheehan KM, Holloway RL, Towne JB. Valuing educational scholarship at the Medical College of Wisconsin. *Acad Med*. 2000;75(9):930-4.

14. Trimm F, Caputo G, Bostwick S, Frohna J, Haftel H, Waggoner-Fountain L, Li ST. Developing leaders in pediatric graduate medical education: the APPD LEAD Program. *Acad Pediatr*. 2015;15(2):143-6.

15. Walvoord E, Hobson-Rohrer W, Bogdewic S. Getting promoted: turning your clinical work into scholarship. *MedEdPORTAL*. 2014; 10, 9694.

16. Bonifacino E, Ufomata EO, Farkas AH, Turner R, Corbelli JA. Mentorship of Underrepresented Physicians and Trainees in Academic Medicine: a Systematic Review. *J Gen Intern Med*. 2021;36(4):1023-1034.

17. Zambrana RE, Ray R, Espino MM, Castro C, et al. “Don’t Leave Us Behind”: The Importance of Mentoring for Underrepresented Minority Faculty. *Am Educ Res J*. 2015; 52(1): 40–72.
