# Supplementary material for: Fostering Success and Promoting Professional Development of Clinician Educator Mentees: A Workshop for Mentors
Source: MedEdPORTAL. 2023 Jun 27;19:11321. doi: 10.15766/mep_2374-8265.11321 (PMC10293477; doi:10.15766/mep_2374-8265.11321)
Supplement: Supplementary file 1 — CE Training Workshop.pptxFacilitator Guide.docxIndividual Development and Mentoring Plans.docxCase Studies.docxResource Guide.docxWorkshop Evaluation.docx [file mep_2374-8265.11321-s001.zip › F. Workshop Evaluation.docx]

**Clinician Educator Mentor Training Module Evaluation***

**SECTION 1: Professional Background**

**1. What is your title?**

- Instructor
- Assistant Professor
- Associate Professor
- Professor
- Other (please specify) ________________________________________________

**2. Which advanced degrees or programs have you completed?** (choose all that apply)

- Masters
- PhD
- MD
- MD/PhD
- DDS
- DVM
- Other (please specify) ________________________________________________

**3. What is your academic advancement “track”?**

- Tenure
- Clinician educator
- Research
- Teaching
- Professional practice or clinical service

**4.  Which category(s) best describes the focus of your research?** (choose all that apply)

- Behavioral research
- Clinical research
- Community engaged research
- Educational research
- Field/Applied research
- Lab-based research (basic)
- Theoretical research
- Translational research (specify type: T1, T2, etc.) ________________________________________________
- Other ________________________________________________

**SECTION 2: Mentoring Experience and Training**

**5.  What kinds of trainees are you currently mentoring?** (choose all that apply)

- Senior faculty
- Junior faculty
- Postdoctoral fellows
- Clinical fellows
- PhD students or masters students
- Medical or health care professional students
- Post-baccalaureate students
- Undergraduates
- High school students
- K awardees
- T awardees
- I am not currently mentoring trainees

**6. In what context is most of your mentoring?** (choose all that apply)

- Education
- Clinical service
- Career advancement
- Research
- Work-life integration
- Networking
- Other ________________________________________________

**7. How many years of experience do you have as a formal mentor?**

________________________________________________________________

**8. Prior to the training you just completed, had you ever participated in, or facilitated any formal mentor training before?** (This only includes training that directly addresses the topic of mentoring, not workshops on related themes like communication, career development, etc.)

- Yes (please describe) ________________________________________________
- No

If yes, how many hours had you previously participated in or facilitated formal mentor training (prior to the training you just completed)?

________________________________________________________________

**SECTION 3: Mentoring Skills**

**9.  Please rate how skilled you feel you were BEFORE attending the research mentor training, and how skilled you feel you are NOW in each of the following areas: (Think about your skills generally, with all your mentees. Please only choose 'not applicable' (NA) if a skill cannot be applied to any of your mentees.)**

|  | Not at all skilled 1 | 2 | 3 | Moderately skilled  4 | 5 | 6 | Extremely skilled  7 | N/A |
| --- | --- | --- | --- | --- | --- | --- | --- | --- |
| 7a. Working with mentees to set clear expectations of the mentoring relationship BEFORE |  |  |  |  |  |  |  |  |
| 7b. Working with mentees to set clear expectations of the mentoring relationship NOW |  |  |  |  |  |  |  |  |
| 8a. Aligning your expectations with your mentees’ BEFORE |  |  |  |  |  |  |  |  |
| 8b. Aligning your expectations with your mentees’ NOW |  |  |  |  |  |  |  |  |
| 15a. Motivating your mentees BEFORE |  |  |  |  |  |  |  |  |
| 15b. Motivating your mentees NOW |  |  |  |  |  |  |  |  |
| 16a. Building mentees’ confidence BEFORE |  |  |  |  |  |  |  |  |
| 16b. Building mentees’ confidence NOW |  |  |  |  |  |  |  |  |
| 17a. Stimulating your mentees’ creativity BEFORE |  |  |  |  |  |  |  |  |
| 17b. Stimulating your mentees’ creativity NOW |  |  |  |  |  |  |  |  |
| 18a. Acknowledging your mentees’ professional contributions BEFORE |  |  |  |  |  |  |  |  |
| 18b. Acknowledging your mentees’ professional contributions NOW |  |  |  |  |  |  |  |  |
| 22a. Helping your mentees network effectively BEFORE |  |  |  |  |  |  |  |  |
| 22b. Helping your mentees network effectively NOW |  |  |  |  |  |  |  |  |
| 23a. Helping your mentees set career goals BEFORE |  |  |  |  |  |  |  |  |
| 23b. Helping your mentees set career goals NOW |  |  |  |  |  |  |  |  |
| 24a. Helping your mentees balance work with their personal life BEFORE |  |  |  |  |  |  |  |  |
| 24b. Helping your mentees balance work with their personal life NOW |  |  |  |  |  |  |  |  |
| 25a. Understanding your impact as a role model BEFORE |  |  |  |  |  |  |  |  |
| 25b. Understanding your impact as a role model NOW |  |  |  |  |  |  |  |  |
| 26a. Helping your mentees acquire resources (e.g. grants, etc.) BEFORE |  |  |  |  |  |  |  |  |
| 26b. Helping your mentees acquire resources (e.g. grants, etc.) NOW |  |  |  |  |  |  |  |  |

**10. How would you rate the overall quality of your mentoring you are able to provide thinking back to before the training and now, after the training?**

|  | Very Low  1 | 2 | 3 | Average  4 | 5 | 6 | Very high  7 |
| --- | --- | --- | --- | --- | --- | --- | --- |
| Before the training |  |  |  |  |  |  |  |
| After the training |  |  |  |  |  |  |  |

**11. To what extent do you feel that you are able to meet your mentees' expectations thinking back to before the training and now, after the training?**

|  | Not at all  1 | 2 | 3 | Moderately  4 | 5 | 6 | Completely  7 |
| --- | --- | --- | --- | --- | --- | --- | --- |
| Before the training |  |  |  |  |  |  |  |
| After the training |  |  |  |  |  |  |  |

| Page Break |  |
| --- | --- |

**SECTION 4: Mentor Training Satisfaction**

**12. Overall, how effective were the facilitators in guiding discussion during your mentor training?**

- Very effective
- Effective
- Neither Effective nor Ineffective
- Ineffective
- Very Ineffective

**13. Was attending this training a valuable use of your time?**

- Yes
- No

**14. How likely are you to recommend the training to a colleague?**

- Very likely
- Likely
- Undecided
- Unlikely
- Very Unlikely

**15. Please rate how skilled you feel you were BEFORE the workshop and how skilled you feel you are NOW in each of the following areas:**

|  | Not at all skilled  1 | 2 | 3 | Moderately skilled  4 | 5 | 6 | Extremely skilled  7 |
| --- | --- | --- | --- | --- | --- | --- | --- |
| Defining demonstrable markers of progress towards career advancement on the Clinician educator career track - BEFORE |  |  |  |  |  |  |  |
| Defining demonstrable markers of progress towards career advancement on the Clinician educator career track - NOW |  |  |  |  |  |  |  |
| Developing a strategy for guiding the professional development of Clinician educator mentees using some form of written document- BEFORE |  |  |  |  |  |  |  |
| Developing a strategy for guiding the professional development of Clinician educator mentees using some form of written document- NOW |  |  |  |  |  |  |  |
| Prioritizing tasks which will lead to advancement/ promotion (including possibility of tenure) and empower mentees to ask for what they need to succeed -BEFORE |  |  |  |  |  |  |  |
| Prioritizing tasks which will lead to advancement/ promotion (including possibility of tenure) and empower mentees to ask for what they need to succeed - NOW |  |  |  |  |  |  |  |
| Identifying resources for mentoring Clinician educators - BEFORE |  |  |  |  |  |  |  |
| Identifying resources for mentoring Clinician educators - NOW |  |  |  |  |  |  |  |

**16. Please rate the usefulness of each training element:**

|  | Not at all useful  1 | 2 | 3 | Moderately useful  4 | 5 | 6 | Extremely useful  7 |
| --- | --- | --- | --- | --- | --- | --- | --- |
| Group discussions |  |  |  |  |  |  |  |
| Case studies |  |  |  |  |  |  |  |
| IDPs |  |  |  |  |  |  |  |
| Mentoring Resources |  |  |  |  |  |  |  |
| Overall |  |  |  |  |  |  |  |

**17. Have you made any, or do you plan to make any changes in your mentoring as a result of this training?**

- Yes
- No

**18.  Please describe any changes you have made in your mentoring, or plan to make as a result of this training.**

________________________________________________________________

________________________________________________________________

________________________________________________________________

________________________________________________________________

________________________________________________________________

**19. What are the strengths and weaknesses of this mentor training? Are there things you would like to see changed or added?  If so, what?**

________________________________________________________________

________________________________________________________________

________________________________________________________________

________________________________________________________________

________________________________________________________________

**20. Any additional comments about the training?**

________________________________________________________________

________________________________________________________________

________________________________________________________________

________________________________________________________________

________________________________________________________________

**SECTION 5: Demographics**

**Are you Hispanic or Latino?**

- No I am not Hispanic or Latino
- Yes I am Cuban
- Yes I am Mexican or Chicano
- Yes I am Puerto Rican
- Yes I am Other Hispanic or Latino (please specify) ________________________________________________
- Prefer not to report

**With which race(s) do you identify?** (choose all that apply)

- American Indian or Alaskan Native
- Asian
- Black or African American
- Native Hawaiian or Pacific Islander
- White
- Other (please specify) ________________________________________________
- Prefer not to report

**Which of the following best describes your gender identity?** (choose all that apply)

- Male
- Female
- Transgender
- Intersex
- Other (please specify) ________________________________________________
- Prefer not to report

**Thank you for completing this survey!**

*Note this survey was run on Qualtrics
