# Supplementary material for: Fostering Success and Promoting Professional Development of Clinician Educator Mentees: A Workshop for Mentors
Source: MedEdPORTAL. 2023 Jun 27;19:11321. doi: 10.15766/mep_2374-8265.11321 (PMC10293477; doi:10.15766/mep_2374-8265.11321)
Supplement: Supplementary file 1 — CE Training Workshop.pptxFacilitator Guide.docxIndividual Development and Mentoring Plans.docxCase Studies.docxResource Guide.docxWorkshop Evaluation.docx [file mep_2374-8265.11321-s001.zip › B. Facilitator Guide.docx]

**Learning Objectives for Fostering Success and Promoting Professional Development of Your Clinician Educator Mentees: A Workshop for Mentors**

**Mentors will have the knowledge and skills to:**

1. Define demonstrable markers of progress toward career advancement on the clinician educator track
2. Develop a strategy for guiding the professional development of clinician educator mentees using an individual development plan
3. Prioritize tasks that will lead to advancement / promotion
4. Access an enhanced toolbox of resources for mentoring clinician educators

**Overview of Activities for the Session:**

|  | **Learning Objectives** | **Core Activities** |
| --- | --- | --- |
| 1 | Define demonstrable markers of progress toward career advancement on the clinician educator track | Activity #1:  Identify and define concrete markers of success and timelines for career advancement (**Appendix A and B**) |
| 2 | Develop a strategy for guiding the professional development of clinician educator mentees using an individual development plan | Activity #2:  Mentors review Individual Development Plans (IDPs) adapted for clinician educators (**Appendix C**)  Activity #3:  Mentors discuss how to use the IDPs reviewed in Activity #2 as a guide for conversations with their mentee about career development |
| 3 | Prioritize tasks that will lead to advancement/promotion | Activity #4:  Mentors review and discuss one of three case studies (**Appendix D**) |
| 4 | Access an enhanced toolbox of resources for mentoring clinician educators | Activity #5:  Share resources for career advancement of clinician educators and for mentoring (**Appendix E**) |

**Materials needed**:

- White board /flip chart, dry erase or permanent marker
- Notecards for concluding commitment exercise
- Pre-print **Appendices C, D, E** for distribution during the workshop. Note that **Appendix D** should not be printed as a whole packet together but as individual cases for discussion.
- Appendix A PowerPoint presentation (and ability to display) to guide the following activities
- Piece of paper asking for names/email addresses on sign in to send **Appendix E** (resources) at end of workshop if not printing out and **Appendix F** (evaluation) if not printing out and doing on site

**Notes to Facilitators:**

***BEFORE*** starting this workshop, facilitators must really know the criteria for clinician-educator promotion at their own institution.

It is helpful to have more than one facilitator for this workshop, particularly if the group is more than 10 people.

**Facilitator roles** (depending on the number of facilitators you have, each person can play more than one role): timekeeper, recorder (for large group discussion, writing answers on flip charts or whiteboard), leading the discussion for each section, overseeing small group/pair discussions.

**Time keeping:** This workshop engenders *a lot* of conversation so keeping participants on task is important. It may be helpful to acknowledge this by saying at the beginning of the workshop: “We know this topic usually provokes lively discussion, which is what we love. However, due to time constraints there may be points at which we have to limit the conversation and hope that you will carry on the discussion when you leave here.”

For each of the discussion questions there are *sample* answers provided based on experience giving this workshop previously. These are not all-inclusive but may be used as a prompt if no one is answering or potentially to fill in any gaps that may arise in discussion.

**Introduction:** (Use **Appendix A**, PowerPoint presentation) (5 minutes)

1. Ask participants to provide their emails on a sheet of paper at the table
2. Icebreaker of your choice (Slide 2)
3. Outline objectives for workshop: BEFORE you read the objectives, to help you get to know your audience, ask the following questions: (Slide 3)
   1. How many of you have a mentee or more than one mentee?
   2. How many of your mentees are clinician educators?
   3. Do you have mentees outside your section? Outside your department? Institution?

**Objective 1: Help mentees define demonstrable markers of progress toward career advancement on the clinician educator career track** (20 min)

**ACTIVITY #1:** Defining Markers of Success (20 min)

ASK (10 min) in the large group: You may want to record the ideas generated in this discussion on a whiteboard or flip chart, writing the markers of success and noting where participants disagree.

1. What does it take, in general, to be a successful clinician educator? (Slide 4)
   1. Evidence of outstanding teaching based on evaluations/awards
   2. Evidence of innovative curricular development/teaching methods
   3. Dissemination of educational products
2. What are the markers of success? (Slide 4)
   1. Peer-reviewed publications
   2. Invited presentations or workshops at national meetings
   3. Attainment of academic leadership role (e.g., program director)
   4. Membership on national professional society committees
   5. Attaining the rare educational grant!
   6. Curricular innovations including new teaching modalities and assessments
   7. Dissemination of curricular innovations via MedEdPORTAL sites, or other technology
   8. Adaptation of curricular innovations by other institutions
   9. Participation in accreditation activities (e.g., American Board of Internal Medicine, Liaison Committee on Medical Education, Accreditation Council for Graduate Medical Education, etc.)
3. What counts and does not count for success in your institutional context? How do you find out if you don’t know? (Slide 4)
   1. Refer to what the clinician educator track is and is not in your institutional context when engaging mentors in this exercise.
   2. If other career pathways come up in discussion, use a parking lot for those elements of advancement/markers of success so that participants feel heard, but you keep them focused on the clinician educator career track.
   3. Know your institutional faculty affairs personnel, chair of the promotions committee, website for your institution that outlines promotions criteria as potential resources

DISCUSS (10 min) in a large group some of the following questions:

Discussion Points for Facilitators: Please note you may not get to all of these questions. This is often dependent on the energy of your group, their level of experience etc. So be flexible in what you get to BUT it is important for the rest of the workshop to try to get to questions 3 and 4. We suggest limiting discussion of questions 1 and 2 to a total of 5 minutes.

1. How can you determine if someone is a successful clinician educator? Is there a certain level of success (or area of mastery) that needs to be achieved? (Slide 5)
   1. You’ve discussed above what generally it looks like to be a successful clinician educator in general. In the answers to this question participants may state that there are different levels of mastery depending on the level of promotion. Examples include:
      1. Assistant Professor: x number of publications, local (within the region or institution) dissemination, educational innovation
      2. Associate Professor: x number of publications, evidence of regional and national dissemination, evidence of educational innovation as measured by uptake of curriculum, mentorship tables
      3. Professor: x number of publications, national recognition or involvement in professional societies, national dissemination, grants (if possible, in education-difficult to obtain)
2. Do markers of success/mastery differ among institutions? Among departments/units?  Are any markers constant? (Slide 5)
   1. How participants answer this question may be dependent on if everyone in the workshop is from the same institution.
      1. Allow discussion of different criteria at different institutions
      2. If from the same institution then is everyone from the same Department? If not, then discuss differences among Departments
      3. Answers will be institution specific so very helpful that facilitators know the promotion criteria for CEs in their institution BEFORE starting this workshop ( as mentioned above)

NOTE: For Questions 3 and 4 first ASK participants to think of one or two of their current mentees, both those they consider very successful and those that may be struggling. Then before you read each of the questions out loud say “I want you to keep those mentees firmly fixed in your mind as we look at this next set of questions. This workshop should help you to answer them but it’s important to acknowledge both their role and yours in this mentoring relationship as we move forward.”

1. Do mentees know what level of mastery is expected of them? (Slide 5)
   1. The answers are often “yes”, “no” I don’t know” or “I haven’t thought to ask.”
   2. You can ask a follow up question of “How do you know?” if you have time.
2. Do you think your mentee’s estimations of their level of mastery are aligned with yours? (Slide 5)
   1. The answers are often “yes”, “no” I don’t know” or “I haven’t thought to ask.”
   2. You can ask a follow up question of “how do you know?” if you have time.
3. Is there ever a point in the mentoring relationship in which the mentee is so independent that they no longer need the mentor? (Slide 5)
   1. They may no longer need you as a mentor, but they may still need a mentor
   2. Sure, they may be senior enough that now they’re mentoring others.
   3. Maybe I could consider them a peer mentor now?

FOLLOW-UP ACTIVITY (Let participants know that there is some “homework” they are encouraged to do outside of the workshop): “One of the things we’re going to ask throughout this workshop is to do some homework. You don’t get off the hook once you leave here! There are some follow up activities and we would love to know how this works for you. The first homework is to draw your own timeline for establishing markers of success/ mastery and discuss it with your mentee to see if it aligns with their expectations.”

**Objective 2: Develop a strategy for guiding the professional development of clinician educator mentees using an individual development plan** (20 min)

**ACTIVITY #2:** Reviewing Individual Development Plans (IDP) and Mentoring Plans (10 min) (Slide 7)

Facilitators: hand out **Appendix C** (**Individual Development Plans**) and ask participants to review individually. As you are handing it out ASK: “Does anyone know what an Individual Development Plan or IDP is?” If participants indicate they know what it is then ask “Does anyone uses an IDP specifically with their CE mentees?” (IDPs are frequently used with research mentees but they are not as specifically used with CEs.)

REVIEW (10 min) individually: Mentors review example plans for Clinician Educators (**Appendix C)** individually. ASK participants to make notes on them to indicate which aspects of the plans they would like to adopt for use with their own mentees. Some mentors may already use such plans and may wish to share additional suggestions.

**ACTIVITY #3:** Using the Individual Development Plans and Mentoring Plans (10 min) (Slides 8-9)

TELL: It is critical to initiate and sustain periodic conversations with mentees on professional goals and career development objectives and strategies.

ACTIVITY (10 min) in pairs: ASK participants to share specific ways they could introduce the idea of an individual development plan to their mentee and how the completed plan can be used to navigate the mentoring relationship.

Sample answers may include:

Introducing IDPs at the start of a new mentoring relationship

Reviewing IDPs annually

Reviewing when new opportunities arise

Reviewing when mentees are not meeting expectations

FOLLOW-UP ACTIVITY: Mentors should ask their mentee to complete an Individual Development Plan and Mentoring Plan annually (at a minimum). The completed plan should be used to guide a conversation with mentor and mentee about professional development needs, barriers, and expectations.  This is a useful activity to periodically realign expectations/goals.

**Objective 3. Prioritizing tasks which will lead to advancement/promotion** (25 min)

**ACTIVITY #4:** (25 min) (Slides 12-18)

One facilitator should be handing out **Appendix D** (**Case studies**) as you introduce the cases. Have enough copies of each case for every participant. Cases should be printed individually, not in a whole packet.

The discussion for the case studies can be done in small groups or a large group depending on the number of participants/facilitators. The larger the group (>10 participants, minimum 2 facilitators) it is preferred to be done in groups of no more than 5 participants.

ASK (5 mins) participants to individually review one of the three case studies provided. Based on previous group discussions you may choose the case or allow the group to choose which case to discuss. The other cases are available if you have additional time. (**Appendix D**)

DISCUSS (10 mins) in the large group the guiding questions for discussion

**Case 1: Yes, Yes, Yes (but no follow through) (Slides 12-13)**

1. What are the main themes raised in this case? Below are some common themes but many more may come out as well.
   1. Little protected time
   2. Doesn’t know how to say no
   3. Not picking one project to take to completion
   4. Time management skills
   5. Mentor isn’t always aware of the barriers
2. How can you help Dr. K. to negotiate and manage the time?
   1. Pick one project and follow through to completion
   2. Work by doing “baby steps” figuring out what part of a project could be done in 30 minutes versus 60 etc. and fitting in the pieces as time becomes available
   3. When asked to do a new project asking Dr. K. to say “I’ll let you know next Monday if I can take this on”. This statement gives Dr. K. time to reflect if Dr. K. can do it and/if something else needs to come off Dr. K.’s plate in order to do it
   4. Ask Dr. K. to reflect if the new project is a “have to” or a “want to”
   5. Ask Dr. K. if new project is in line with the area of content Dr. K. wishes to develop in Dr. K.’s career
   6. Work with Dr. K. to gracefully learn to say no to projects
3. What steps would you take to hold yourself and Dr. K. accountable in the future?
   1. Set deadlines for pieces of projects, not the whole project
   2. Make steps manageable
   3. You as the mentor need to follow through with email or a meeting to see where Dr. K. is in meeting goal. You may need to set a reminder on your own calendar. You can also email Dr. K. a calendar appointment of the deadline.
   4. You or the mentee should send a summary email of your meeting and expectations for the next meeting
   5. Make sure there’s an agenda for each meeting (set by mentee ideally)
   6. Find out what the barriers are when expectations are not met
4. What would be your threshold for renegotiating the terms of this mentoring relationship, including no longer serving as the primary mentor, time spent, etc.? Renegotiation will be specific to the mentor/mentee.
   1. Ask mentee (particularly if goals consistently still not being met) how they think you, as the mentor, can continue to help
   2. State maybe you are not the right mentor and offer to suggest someone else or introduce mentee to someone else who might be a better fit
   3. Make conversation behavioral and not personal

**Case 2: Complain, complain, complain. Faculty who wants to get promoted but never takes your suggestions** (Slides 14-15)

1. What are the main themes raised in this case?
   1. No protected time and heavy clinical load
   2. Time management (both of clinical work and non-clinical)
   3. More of an “idea” person maybe not as much of a detail or execution person
   4. Externalizing by the mentee (blaming the problem on “the system” but not seeing what role Dr. S. plays in the lack of forward progress)
2. How would you define the problem(s) with which Dr. S. presents you?
   1. Lack of clarity on what Dr. S. really wants (both in your role as mentor and Dr. S. as faculty member)
   2. Lack of focus (does Dr. S. want to be in QI/education/clinical innovation?)
   3. Not a good use of mentoring time
   4. Maybe you are not a good mentor/mentee fit
3. How would you guide Dr. S. towards fine-tuning a set of career goals?
   1. Ask to write a list of what Dr. S. love/ dislikes about the job, seeing where is the balance
   2. Help identify areas of overlap with Dr. S.’s clinical work so there is synergy between work and scholarship (e.g., is there a quality metric on which Dr. S scores poorly so Dr. S. could lead a quality project to improve individually and as a clinic?)
4. How do you help mentees identify alternative career paths to academic medicine?
   1. Ask why they joined an academic practice initially and what has changed,
   2. Ask about other skills (e.g., information technology) to see if there are other roles that might be a better fit
   3. Connect them with others in different career paths or in private practice
   4. Talk with the advisory/student affairs dean at your institution about where graduates have gone if not into clinical medicine

**Case 3: Stuck at Assistant Professor. Great educator who seems stuck at Assistant Professor** (Slides 16-17)

1. What are the main themes raised in this case?
   1. Depending on the pathway/institution publications often a barrier for promotion for busy CE (other forms of dissemination not weighing as much)
   2. Focus has been on teaching, not dissemination
   3. How does the institution value teaching
   4. How to demonstrate value of work being down
   5. Lack of training in educational design, evaluation, writing
2. In the context of your institutional criteria for promotion, what are the specific barriers to promotion that Dr. A. faces?
   1. This is very institution dependent so important to know your institutional criteria well.
   2. Barriers may include
      1. No appropriate pathway for promotion
      2. Emphasis on publications/dissemination, criteria for certain position (e.g., Associate Dean role)
3. What strategies have you seen successful clinician educators pursue that you may be able to suggest to Dr. A. in working toward promotion?
   1. Collaborating with others on projects, particularly those who are good at publishing
   2. Having educational learning groups in which all involved work on different parts of project/paper and get multiple publications
   3. Involvement in regional/national societies
   4. Schedule specific time on calendar to work on project
4. How would you help Dr. A. outline a strategy for promotion? What resources are available from your department/school/university to assist the process?
   1. Review CV and see weak points on which focus, discuss strategies from Question 3.
   2. Resources: This is institution specific
      1. Head of the promotions committee
      2. Faculty who have been successfully promoted in the CE track
      3. Division chiefs/ department chairs
      4. Faculty affairs office
      5. Institutional website with promotion criteria

DISCUSS (10 mins) in the large group the following questions:

1. What approaches do you currently use to help your mentee(s) navigate career decision?
   1. Having a mentoring team (project content/methods mentors often different from career mentors)
   2. Encouraging meeting with mentors outside division/department/institution
   3. Lists of pros/cons about current role
2. What approaches might you consider using going forward?
   1. IDP
   2. Mentoring meeting summaries
   3. CV review annually (with mentor or other faculty resource)

**Objective 4: Access an enhanced toolbox of resources for mentoring Clinician Educators**

ACTIVITY #5: (5 minutes) (Slide 19)

TELL: Hand out **Appendix E** (**packet of resources)**. Indicate that you will share this electronically if everyone has put down their email address on the paper at their table.

Discuss briefly what other resources participants might use that are not included.

- 1. Ask participants if anyone has any other resources that they like to share?
  2. Point out that this is a living document. The AAMC updates their places to publish journals every two to three years.
  3. Let participants know that the resource packet includes links to overall societies, not specific meetings as these change from year to year.

**Conclusion:** (15 minutes) (Slides 20-21)

Timing is 5-6 minutes for commitment/pair share, rest of time for evaluation

1. ASK participants to write on the notecard provided a commitment to one new practice that they will implement in their clinician educator mentoring moving forward
2. Have participants share these with the large group or with a partner depending on the size of the group
3. Answer final questions
4. Deploy **CE Mentor Training Evaluation** (**Appendix F**) either in the workshop or afterwards. ASK participants to fill out paper copies of survey provided or indicate you will send a Qualtrics or similar survey software version depending on how you decide to deploy your evaluation. If you decide to do electronically then you may have options for QR code or electronic link to survey depending on your institutional software.
