# Supplementary material for: Fostering Success and Promoting Professional Development of Clinician Educator Mentees: A Workshop for Mentors
Source: MedEdPORTAL. 2023 Jun 27;19:11321. doi: 10.15766/mep_2374-8265.11321 (PMC10293477; doi:10.15766/mep_2374-8265.11321)
Supplement: Supplementary file 1 — CE Training Workshop.pptxFacilitator Guide.docxIndividual Development and Mentoring Plans.docxCase Studies.docxResource Guide.docxWorkshop Evaluation.docx [file mep_2374-8265.11321-s001.zip › Educational Summary Report_11321.pdf]

MedEdPORTAL® The AAMC Journal of Teaching and Learning Resources

Original Publication

Open Access

Fostering Success and Promoting Professional Development of Clinician Educator Mentees: A Workshop for Mentors

Hollis D. Day, MD, MS, MHPE*, Emelia J. Benjamin, MD, ScM, Sangeeta Lamba, MD, MS-HPEd, Keith C. Norris, MD, PhD, Christine Pfund, PhD, Maria L. Soto-Greene, MD, MS-HPEd

*Corresponding author: Hollis.Day@bmc.org

Abstract

Introduction: The goal of academic mentoring relationships is to enable the mentee to identify/achieve professional advancement. Although mentors of clinician educators (CEs) must understand the criteria for successful career advancement, few have received formal CE mentor training. **Methods:** The National Research Mentoring Network convened an expert panel to develop a 90-minute module for training CE mentors. This module included individual development plans, case studies involving challenges for CE faculty, and examples of the broadened scope of scholarly activity. The workshop was delivered to 26 participants across four institutions and evaluated by a retrospective pre/post survey. **Results:** Using a 7-point scale (1 = *very low,* 4 = *average,* 7 = *very high*), participants rated the overall quality of their CE mentoring as slightly below average preworkshop ($M = 3.9$) and as above average postworkshop ($M = 5.2$, $p < .001$). Areas of greatest self-perceived change in skills on a 7-point scale (1 = *not at all skilled,* 4 = *moderately skilled,* 7 = *extremely skilled*) included setting clear expectations of the mentoring relationship (pre $M = 3.6$, post $M = 5.1$, $p < .001$), aligning mentor expectations with those of mentees (pre $M = 3.6$, post $M = 5.0$, $p < .001$), and helping mentees set career goals (pre $M = 3.9$, post $M = 5.4$, $p < .001$). **Discussion:** This module trains CE mentors using an interactive and collective problem-solving approach. Workshop participants better defined demonstrable markers for CE progression with potential to impact tailored guidance for mentees.

Keywords
Clinician Educator, Case-Based Learning, Faculty Development, Mentoring/Coaching, Promotions & Tenure

Educational Objectives

By the end of this workshop, learners will be able to:

1. Define demonstrable markers of progress toward career advancement on the clinician educator track.
2. Develop a strategy for guiding the professional development of clinician educator mentees using an individual development plan.
3. Prioritize tasks that lead to advancement/promotion.
4. Access an enhanced toolbox of resources for mentoring clinician educators.

Citation:
Day HD, Benjamin EJ, Lamba S, Norris KC, Pfund C, Soto-Greene ML. Fostering success and promoting professional development of clinician educator mentees: a workshop for mentors. *MedEdPORTAL.* 2023;19:11321. https://doi.org/10.15766/mep_2374-8265.11321

Introduction

One goal of academic mentoring relationships is to enable the mentee to identify/achieve professional outcomes required for career advancement. Defining and demonstrating progress towards the mentee's academic objectives and explaining how the objectives contribute to the establishment of a unique identity or area of mastery are critical elements of success that mentors help their mentees navigate. For the purposes of this workshop, we define clinician educators (CEs) as faculty who are practicing clinicians and teaching either in the classroom or in the clinical context.

As academic medicine has evolved, so have metrics of success for promotion across the differing academic tracks. Senior mentors including division chiefs, department chairs, and promotions committee chairs are frequently from clinician scientist pathways, where mentoring programs, infrastructure, and metrics are better established than those for CEs.[1] A review of CE promotion criteria revealed that only 43% of U.S. medical schools had a defined CE promotion pathway and that less than 10% had fully adopted published evaluation/promotion criteria.[2]

Copyright © 2023 Day et al. This is an open-access publication distributed under the terms of the Creative Commons Attribution license.

This deficit in clear promotion criteria potentially leaves CEs being evaluated for academic advancement based on incomplete or unclear criteria and may leave mentors struggling to properly advise CEs.

Mentorship is critical for career development in the clinician scientist pathway, but CEs in one study identified an education mentor less than 33% of the time, and the absence of a mentor was felt to have a negative career impact.[3-5] While the literature has provided frameworks[6] and recommendations for mentorship skills,[7,8] there are fewer opportunities for mentors to enhance their skills at mentoring CEs.[9]

Promotion as a CE is based on excellence in clinical practice, teaching, and service. Institution-specific markers of excellence necessary for advancement/promotion may include excellence in meeting expectations commensurate with academic rank, regional/national reputation based on opinions from senior faculty members, and productivity commensurate with the service portion (clinical, administrative, educational) of one's effort. Frequently, service to the institution is expected and can be time consuming, but the amount of service work that is valued for advancement varies across institutions.

Scholarship and dissemination are expected of a successful CE. We define scholarship broadly and more consistently with Boyer's model of scholarship including domains of discovery, integration, application, and teaching.[10] While CEs may play an integral role as contributors to team science (discovery), more commonly their advancement is assessed in integration (e.g., comprehensive reviews/textbook chapters), application (e.g., leadership in professional societies), and teaching. Teaching may occur in multiple settings, including classroom, online, and clinical sites, and is traditionally assessed by teaching evaluations. Learners range from students, residents, and fellows to faculty and nonacademic clinicians.

CEs are frequently expected to demonstrate dissemination of their efforts as evidenced by published curricula, papers, *MedEdPORTAL* resources, invited talks, and conference workshops. Other forms of expected recognition include membership/leadership in regional/national academic societies or program review, certification/accreditation committees, and so on.

Although mentors of CEs must understand the academic and professional criteria for successful career advancement, few have received formal training in mentoring CEs.[3] To address this need, in 2018 the National Research Mentoring Network (NRMN) convened an expert panel composed of this publication's authors to develop and pilot an interactive module for training mentors of CEs. The resulting module (henceforth, the CE Mentor Training Module) was designed as a freestanding tool to train mentors of CEs using an interactive and collective problem-solving approach. It expands upon existing training for research mentors that includes aligning expectations, culturally aware mentoring, and self-efficacy.[8]

Methods

The curriculum development team (the authors) obtained institutional review board approval through NRMN protocols 2019-0956, 2015-0871, and 2016-0458 and Center for the Improvement of Mentored Experiences in Research protocol 2017-1336. The authors were researchers and CEs with extensive CE mentoring experience. To develop workshop content, we searched PubMed using the terms *clinician educator, clinical educator,* and *mentoring* and restricted articles reviewed to the English language. We reviewed *MedEdPORTAL* content and noted resources that taught mentoring skills or were directed at specific training levels, for example, medical students or residents.[9,11] Furthermore, those of us who had participated in NRMN workshop experiences cited frequent questions about mentoring CEs as a practice gap.

Existing content in the evidence-based Entering Mentoring mentor training curriculum was organized into roughly 1-hour stand-alone modules covering a variety of topics (maintaining effective communication, aligning expectations, assessing understanding, addressing equity/inclusion, fostering independence, promoting professional development, articulating a mentoring philosophy).[8] We developed content and integrated the CE Mentor Training Module into the structure of the Entering Mentoring curriculum, as well as developing brief didactic material on identifying institution-specific promotion criteria for CEs with large-group discussion (Appendix A) and a companion facilitator's guide (Appendix B). The result was a freestanding, 90-minute, interactive, in-person module.

We served as facilitators at our four respective institutions. We delivered the workshop to 26 participants across these institutions as a stand-alone module, except in one case where the workshop complemented previously implemented mentorship educational sessions. We delivered the session once at each site during preexisting faculty development conference time, with four to 12 participants depending on the site. We used two facilitators per workshop. Workshop participants were mentors of early-career stage CEs. In addition to materials

Copyright © 2023 Day et al. This is an open-access publication distributed under the terms of the Creative Commons Attribution license.

provided in the appendices, resources included a whiteboard/flip chart with markers for group discussion, note cards for participant commitments, and ability to display a PowerPoint presentation.

After outlining institution-specific promotion criteria and general markers of success in the large group, each participant reviewed examples of individual development plans (IDPs; Appendix C). In pairs, participants discussed introducing IDPs to mentees and using them to navigate the mentoring relationship. Utilizing knowledge gained from the first two portions of the workshop, participants then broke into small groups to practice overcoming known challenges in the CE mentoring relationship via case studies (Appendix D). We provided participants with a list of resources including sample journals accepting educational scholarship, articles on writing educational grants, potential sources of funding, and conferences for dissemination of medical education scholarship (Appendix E). At the conclusion of the workshop, participants were invited to complete the workshop evaluation form (Appendix F) via Qualtrics.

Nineteen of 26 participants completed a retrospective pre/post survey[12] to evaluate the workshop. We performed a matched *t* test with significance set at $p < .01$ for data collectively from all sites as the number of participants at each individual site was too small to determine a meaningful change. We calculated Cohen's effect size. Also, we collated the open comments in the evaluation and surveyed for themes.

Results

Of the 19 respondents, 73% were assistant professors, 8% were associate professors, and 15% were at the instructor level; 4% self-identified as Black, 19% as Hispanic/Latinx, and 66% as White. Sixty-nine percent reported they were on the CE track, acknowledging that nomenclature for CE tracks varied institutionally. Furthermore, 73% of participants had never participated in research mentoring prior to this workshop.

We delivered the workshop across four geographically diverse institutions with consistent results among the 19 participants (73%) who responded to the postworkshop survey. On a 7-point Likert-type scale (1 = *very low,* 4 = *average,* 7 = *very high*), participants rated the overall quality of their mentoring of CEs as slightly below average preworkshop ($M = 3.9$, $SD = 1.2$) and as above average postworkshop ($M = 5.2$, $SD = 0.9$), $t(24) = 1.3$, $p < .001$. Cohen's effect size ($d = 0.74$) suggested moderately high impact. These findings supported our hypothesis that mentors lack formal CE-targeted mentor training in their career paths, a gap this curriculum begins to fill.

We asked participants about mentoring skills using a 7-point Likert-type scale (1 = *not at all skilled,* 4 = *moderately skilled,* 7 = *extremely skilled*). Areas of greatest self-perceived change in skills included working with mentees to set clear expectations of the mentoring relationship (pre $M = 3.6$, $SD = 1.3$; post $M = 5.1$, $SD = 1.2$), $t(24) = 7.9$, $p < .001$; aligning mentor/mentee expectations (pre $M = 3.6$, $SD = 1.2$; post $M = 5.0$, $SD = 1.2$) $t(24) = 7.3$, $p < .001$; and helping mentees set career goals (pre $M = 3.9$, $SD = 1.3$; post $M = 5.4$, $SD = 1.2$) $t(24) = 3.9$, $p < .001$. Cohen's effect ($d = 0.96$) indicated a large effect size. Perceived changes in all areas on the evaluation form were statistically significant at $p < .001$ except for balancing work and personal life, a topic that was not as explicitly covered in this brief module. All (100%) respondents found the training valuable, 90% would recommend the training to colleagues, and 92% reported planning to make changes to their mentoring.

Qualitative data demonstrated common themes, including mentors' intention to use the IDPs to set concrete goals with mentees and the need to set clear expectations with mentees, supporting the quantitative data. The following was a typical open comment:

> I have a better idea of questions to ask of my mentors and a better idea how to encourage mentees to frame their growth. I simply would have loved to have had this available to me earlier in my career.

Another sample comment was "The most helpful thing for me was the document which offered a description of the work that educators do, providing a guide to discuss mentee's goals in each category." Unique comments included wanting facilitators to review institution-specific promotion criteria aloud and wishing the workshop covered grant writing. In the future, facilitators may choose to state abbreviated versions of their institution's promotion criteria, but our experience has proven that a thorough review of criteria merits an additional faculty development session devoted solely to the topic. Similarly, grant writing is a multifaceted topic that requires other forms of programming.

Discussion

The CE Mentor Training Module is a tool to train mentors of CEs using an interactive and collective problem-solving approach. It expands upon existing training for research mentors that includes aligning expectations, culturally aware mentoring, and self-efficacy. This workshop was created based on literature review

Copyright © 2023 Day et al. This is an open-access publication distributed under the terms of the Creative Commons Attribution license.

and expert input and uses methods such as review of IDPs and case studies to enhance mentoring skills.

Typically, mentoring others becomes an increasingly important activity as one advances in academic rank. While the majority of our participants were assistant professors, we did not distinguish between early versus late assistant professor stage. Importantly, at some institutions, due to their promotion criteria/pathways, some faculty remain at rank for extended periods and do mentor more early-career faculty. Regardless of time spent in rank, it was clear from the evaluation data that many mentors felt unprepared for their roles.

We designed our training for any faculty member who mentors CEs. However, most of the participants were themselves on the CE or teaching pathway.

While there were multiple areas of growth noted, the greatest self-perceived changes in skills concerned articulating and aligning expectations, changes also observed in the randomized controlled study of the Entering Mentoring–based curriculum adapted for the mentors of clinical and translational researchers.[13] The data support the generalizability of the content to a diverse population of faculty mentors. Furthermore, open comments and evaluation data support the idea that participants were better able to define markers of success for CEs, use an IDP, and access a toolbox of resources for mentoring CE faculty. It was more challenging to determine if mentors were truly better able to help mentees prioritize tasks based on the case discussion. Longitudinal follow-up with participants would be useful to better characterize changes in this domain.

Limitations to our results include the small number of participants. Despite our anticipating that most of the audience would be clinician scientists, most participants were themselves on the CE track. We may need to further tailor the workshop for an audience that is not familiar with the CE track/promotion criteria. Faculty participants in our cohort were early- to mid-career faculty, and results may vary for more-senior faculty. Whereas we observed significant gains in self-efficacy, the outcomes were self-reported, an inherent limitation. We did not investigate if mentor training improved mentee-reported quality of mentoring. Notably, no participants to date have been full professors, possibly limiting the generalizability of our results. Lack of participation may reflect the paucity of CEs who are full professors and our workshop recruitment strategy.

We learned several lessons from the multi-institutional implementation of this module. First, communication/promotion about the session often dictates the participants who register for training. CE mentors do come from clinician scientist pathways, so it is important to develop promotional materials that are more inclusive. Second, users should consider whether the module should be delivered stand-alone versus as part of a larger program with companion modules covering topics such as mentoring across differences and other diversity/inclusion considerations. Lastly, a stand-alone module is attractive to mid-career CEs mentoring other earlier-career faculty. The shorter time commitment for this module may have generated even more interest among actual CEs due to their competing time constraints.

Future iterations will target faculty participation at the associate and professor levels, particularly those with significant leadership roles, to ensure their understanding of CE mentoring needs. It would be helpful to pilot this workshop at institutions with larger numbers of senior CE faculty. Furthermore, we recommend the module be included in the portfolio of evidence-based mentorship education. We want to develop an extended workshop incorporating a section on empowering mentees to ask for resources needed for their career advancement. Through broader workshop dissemination, we want to investigate the longitudinal impact on participating mentors. Future work will help us understand who self-identifies as mentors of CEs and how to encourage more faculty to effectively mentor this crucial group. Finally, we are developing content for an additional module that addresses intersectional identities and belonging through the CE lens and what challenges faculty are navigating in their personal /professional spaces.

Appendices

A. CE Training Workshop.pptx
B. Facilitator Guide.docx
C. Individual Development and Mentoring Plans.docx
D. Case Studies.docx
E. Resource Guide.docx
F. Workshop Evaluation.docx

All appendices are peer reviewed as integral parts of the Original Publication.

Hollis D. Day, MD, MS, MHPE: Associate Professor, Department of Medicine, Boston Medical Center/Boston University Chobanian & Avedisian School of Medicine; ORCID: https://orcid.org/0000-0001-8817-5160

Copyright © 2023 Day et al. This is an open-access publication distributed under the terms of the Creative Commons Attribution license.

Emelia J. Benjamin, MD, ScM: Professor, Department of Medicine, Boston Medical Center/Boston University Chobanian & Avedisian School of Medicine; Professor, Department of Epidemiology, Boston University School of Public Health

Sangeeta Lamba, MD, MS-HPEd: Professor, Department of Emergency Medicine, Rutgers New Jersey Medical School; Vice Chancellor for Diversity and Inclusion, Rutgers Biomedical and Health Sciences

Keith C. Norris, MD, PhD: Professor, Department of Medicine, University of California, Los Angeles

Christine Pfund, PhD: Senior Scientist, Wisconsin Center for Education Research and Institute for Clinical and Translational Science, University of Wisconsin–Madison

Maria L. Soto-Greene, MD, MS-HPEd: Professor, Department of Medicine, and Executive Vice Dean, Rutgers New Jersey Medical School

Acknowledgments
Thank you to the following team members who contributed to workshop development: Robina Bhasin, EdM, Penn Foster Education Group; Carol S. Newlon, PhD, Rutgers New Jersey Medical School; and Geeta Singhal, MD, Baylor College of Medicine. Thank you to the Institute for Clinical and Translational Research at the University of Wisconsin for supporting evaluation data collection.

Disclosures
None to report.

Funding/Support
This work was supported in part by the National Institutes of Health (NIH) Common Fund and Office of Scientific Workforce Diversity under three linked awards, RL5GM1189XX, TL4GM1189XX, 1UL1GM1189XX, NRMN U54GM119023, and CEC U54GM119024, administered by the National Institute of General Medical Sciences. The work was conducted by members of the Diversity Program Consortium of the Enhancing the Diversity of the NIH-Funded Workforce Program. Additional support was provided by the Rutgers New Jersey Medical School Hispanic Center of Excellence, Health Resources and Services Administration, through grant D34HP26020; 2R01 HL092577, 1R01HL128914; 1P50HL120163 (Emelia J. Benjamin); the Institute for Clinical and Translational Research (Clinical and Translational Science Awards Program at the National Center for Advancing Translational Sciences), grant 1UL1TR002373 (Christine Pfund).

Dr. Benjamin was partially supported by R01HL092577, 2U54HL120163, and American Heart Association AF AHA_18SFRN34110082.

Dr. Norris is supported in part by NIH research grants UL1TR001881, P30AG021684, U2CDK129496, and P50MD017366.

Prior Presentations
Day HD, Bachorik A, Lamba S. Fostering success and promoting development of clinical educator mentees. Workshop presented at: Society of General Internal Medicine National Meeting; April 6-9, 2022; Orlando, FL.

Ethical Approval
Reported as not applicable.

Disclaimer
This work is solely the responsibility of the authors and does not necessarily represent the official view of the National Institutes of Health.

References

1. Geraci SA, Thigpen SC. A review of mentoring in academic medicine. *Am J Med Sci*. 2017;353(2):151-157. https://doi.org/10.1016/j.amjms.2016.12.002
2. Ryan MS, Tucker C, DiazGranados D, Chandran L. How are clinician-educators evaluated for educational excellence? A survey of promotion and tenure committee members in the United States. *Med Teach*. 2019;41(8):927-933. https://doi.org/10.1080/0142159X.2019.1596237
3. Farrell SE, Digioia NM, Broderick KB, Coates WC. Mentoring for clinician–educators. *Acad Emerg Med*. 2004;11(12):1346-1350. https://doi.org/10.1197/j.aem.2004.07.009
4. Shafer KM, Hira RS, Sinha SS, et al. Academic advancement in the current era: integrating and empowering clinician educators. *J Am Coll Cardiol*. 2019;73(5):620-623. https://doi.org/10.1016/j.jacc.2018.11.032
5. Mylona E, Brubaker L, Williams VN, et al. Does formal mentoring for faculty members matter? A survey of clinical faculty members. *Med Educ*. 2016;50(6):670-681. https://doi.org/10.1111/medu.12972
6. Chang A, Schwartz BS, Harleman E, Johnson M, Walter LC, Fernandez A. Guiding academic clinician educators at research-intensive institutions: a framework for chairs, chiefs, and mentors. *J Gen Intern Med*. 2021;36(10):3113-3121. https://doi.org/10.1007/s11606-021-06713-9
7. Kirsch JD, Duran A, Kaizer AM, Thompson Buum H, Robiner WN, Weber-Main AM. Career-focused mentoring for early-career clinician educators in academic general internal medicine. *Am J Med*. 2018;131(11):1387-1394. https://doi.org/10.1016/j.amjmed.2018.07.028
8. Pfund C, House S, Asquith P, Spencer K, Silet K, Sorkness C. *Mentor Training for Clinical and Translational Researchers*. WH Freeman; 2012.
9. Welch J, Palmer M, Mitchell A, et al. Faculty mentoring workshop. *MedEdPORTAL*. 2014;10:9778. https://doi.org/10.15766/mep_2374-8265.9778
10. Kern B, Mettetal G, Dixson M, Morgan RK. The role of SoTL in the academy: upon the 25th anniversary of Boyer's *Scholarship Reconsidered. J Scholarsh Teach Learn*. 2015;15(3):1-14. https://doi.org/10.14434/josotl.v15i3.13623
11. Fenton K, Kim J, Abramson E, Waggoner-Fountain L, Naifeh M, Li ST. Mentoring resident scholarly activity: a toolkit and guide for program directors, research directors and faculty mentors. *MedEdPORTAL*. 2015;11:10103. https://doi.org/10.15766/mep_2374-8265.10103

Copyright © 2023 Day et al. This is an open-access publication distributed under the terms of the Creative Commons Attribution license.

12. Allen JM, Nimon K. Retrospective pretest: a practical technique for professional development evaluation. *J Ind Teach Educ*. 2007;44(3):27-42.

13. Pfund C, House SC, Asquith P, et al. Training mentors of clinical and translational research scholars: a randomized controlled trial. *Acad Med*. 2014;89(5):774-782. https://doi.org/10.1097/ACM.0000000000000218

Received: September 18, 2022
Accepted: March 16, 2023
Published: June 27, 2023

Copyright © 2023 Day et al. This is an open-access publication distributed under the terms of the Creative Commons Attribution license.
